# Consumption of Processed Meat and Unprocessed Red Meat and Risk of Bladder Cancer: A Pooled Analysis of 30 Cohort Studies

**DOI:** 10.3390/cancers18193203

**Published:** 2026-10-03

**Authors:** Ziyu Wang, Nina Afshar, Allison M. Hodge, Harindra Jayasekara, Stella Koutros, Piet A. van den Brandt, Alicja Wolk, Molin Wang, Tao Hou, Hans-Olov Adami, Agneta Åkesson, Chrisa Arcan, Kimberly A. Bertrand, Marisa Bittoni, David Bogumil, Hui Cai, Amanda J. Cross, A. Heather Eliassen, Jo L. Freudenheim, Gretchen L. Gierach, Edward L. Giovannucci, Wen-Yi Huang, Paula Jakszyn, Rieko Kanehara, Victoria A. Kirsh, Woon-Puay Koh, Candyce H. Kroenke, James V. Lacey, Linda M. Liao, Erikka Loftfield, Chaoran Ma, Loic Le Marchand, Maria Elena Martinez, Anthony B. Miller, Steven C. Moore, Lorelei A. Mucci, Heather M. Munro, Marian L. Neuhouser, Jessica L. Petrick, Jenny N. Poynter, Mark A. Preston, Anna Prizment, Samantha Rees, Kim Robien, Thomas E. Rohan, Sven Sandin, Norie Sawada, Eva S. Schernhammer, Marissa M. Shams-White, Martha J. Shrubsole, Xiao-Ou Shu, Rashmi Sinha, Sabina Sieri, Mingyang Song, Meir J. Stampfer, Rachael Z. Stolzenberg-Solomon, Matthew Triplette, Caroline Y. Um, Kala Visvanathan, Trang VoPham, Fenglei Wang, Renwei Wang, Sophia S. Wang, Ying Wang, Elisabete Weiderpass, Lynne Wilkens, Walter C. Willett, Jian-Min Yuan, Wei Zheng, Nathaniel Rothman, Brigid M. Lynch, Debra T. Silverman, Stephanie A. Smith-Warner, Roger L. Milne

**Affiliations:** 1 Cancer Epidemiology Division, Cancer Council Victoria, Melbourne, Victoria 3002, Australia; 2 Centre for Epidemiology and Biostatistics, Melbourne School of Population and Global Health, The University of Melbourne, Melbourne, Victoria 3010, Australia; 3 School of Public Health and Preventive Medicine, Faculty of Medicine, Nursing and Health Sciences, Monash University, Clayton, Victoria 3168, Australia; 4 Division of Cancer Epidemiology and Genetics, National Cancer Institute, National Institutes of Health, Bethesda, MD 20892, USA; 5 Department of Epidemiology, Maastricht University, 6200 MD Maastricht, The Netherlands; 6 Institute of Environmental Medicine, Karolinska Institutet, 171 77 Stockholm, Sweden; 7 Department of Biostatistics, Harvard T.H. Chan School of Public Health, Boston, MA 02115, USA; 8 Department of Epidemiology, Harvard T.H. Chan School of Public Health, Boston, MA 02115, USA; 9 Channing Division of Network Medicine, Brigham and Women’s Hospital, Harvard Medical School, Boston, MA 02115, USA; 10 Department of Nutrition, Harvard T.H. Chan School of Public Health, Boston, MA 02115, USA; 11 Clinical Effectiveness Research Group, Institute of Health and Society, University of Oslo, 0318 Oslo, Norway; 12 Department of Medical Epidemiology and Biostatistics, Karolinska Institutet, 171 77 Stockholm, Sweden; 13 Department of Nutritional Sciences, College of Health and Human Development, University Park, PA 16802, USA; 14 Slone Epidemiology Center, Boston University, Boston, MA 02118, USA; 15 Division of Medical Oncology, College of Medicine, The Ohio State University, Columbus, OH 43210, USA; 16 The Ohio State University Comprehensive Cancer Center, Columbus, OH 43210, USA; 17 Department of Preventive Medicine, Keck School of Medicine, University of Southern California, Los Angeles, CA 90032, USA; 18 Division of Epidemiology, Department of Medicine, Vanderbilt Epidemiology Center, Vanderbilt-Ingram Cancer Center, Vanderbilt University Medical Center, Nashville, TN 37203, USA; 19 Cancer Screening and Prevention Research Group (CSPRG), Department of Surgery and Cancer, Imperial College London, London W2 1NY, UK; 20 Department of Epidemiology and Environmental Health, School of Public Health and Health Professions, University at Buffalo, Buffalo, NY 14214, USA; 21 Unit of Nutrition and Cancer, Cancer Epidemiology Research Programme, Catalan Institute of Oncology (ICO-IDIBELL), 08908 Barcelona, Spain; 22 Department of Cohort Research, Institute for Cancer Control, National Cancer Center, Tokyo 104-0045, Japan; 23 Ontario Institute for Cancer Research, Toronto, ON M5G 0A3, Canada; 24 Dalla Lana School of Public Health, University of Toronto, Toronto, ON M5T 3M7, Canada; 25 Healthy Longevity Translational Research Programme, Yong Loo Lin School of Medicine, National University of Singapore, Singapore 117456, Singapore; 26 Division of Research, Kaiser Permanente Northern California, Pleasanton, CA 94588, USA; 27 Kaiser Permanente School of Medicine, Pasadena, CA 91101, USA; 28 Division of Health Analytics, Department of Computational and Quantitative Medicine, City of Hope, Duarte, CA 91010, USA; 29 Department of Nutrition, School of Public Health & Health Sciences, University of Massachusetts Amherst, Amherst, MA 01003, USA; 30 Epidemiology Program, University of Hawaii Cancer Center, Honolulu, HI 96813, USA; 31 Department of Family and Preventive Medicine, University of California at San Diego, San Diego, CA 92093, USA; 32 Discovery Sciences, American Cancer Society, Atlanta, GA 30303, USA; 33 International Epidemiology Field Station, Vanderbilt-Ingram Cancer Center, Vanderbilt University Medical Center, Nashville, TN 37203, USA; 34 Division of Public Health Sciences, Fred Hutchinson Cancer Center, Seattle, WA 98109, USA; 35 Division of Epidemiology and Clinical Research, Department of Pediatrics, University of Minnesota, Minneapolis, MN 55455, USA; 36 Department of Urology, Mass General Brigham, Boston, MA 02114, USA; 37 Dana-Farber Cancer Institute, Lank Center for Genitourinary Oncology, Boston, MA 02215, USA; 38 Department of Laboratory Medicine and Pathology, University of Minnesota, Minneapolis, MN 55455, USA; 39 Department of Exercise and Nutrition Sciences, Milken Institute School of Public Health, George Washington University, Washington, DC 20052, USA; 40 Department of Epidemiology and Population Health, Albert Einstein College of Medicine, Bronx, NY 10461, USA; 41 Department of Epidemiology, Center for Public Health, Medical University of Vienna, 1090 Vienna, Austria; 42 Department of Population Science, American Cancer Society, Atlanta, GA 30303, USA; 43 Epidemiology and Prevention Unit, Fondazione IRCCS Istituto Nazionale dei Tumori, Milan 20133, Italy; 44 Department of Epidemiology, Johns Hopkins Bloomberg School of Public Health, Baltimore, MD 21205, USA; 45 Sidney Kimmel Comprehensive Cancer Center, Johns Hopkins, Baltimore, MD 21287, USA; 46 University of Pittsburgh Medical Center (UPMC) Hillman Cancer Center, Pittsburgh, PA 15232, USA; 47 Department of Epidemiology, School of Public Health, University of Pittsburgh, Pittsburgh, PA 15261, USA; 48 International Agency for Research on Cancer (IARC), CEDEX 07 69366 Lyon, France; 49 Cancer Center, University of Hawaii, Honolulu, HI 96813, USA; 50 Precision Medicine, School of Clinical Sciences at Monash Health, Monash University, Clayton, Victoria 3168, Australia

**Keywords:** bladder cancer, processed meat, unprocessed red meat, dietary intake, prospective cohort studies, pooled analysis

## Abstract

**Background**: Processed meat and unprocessed red meat are considered carcinogenic and ‘probably carcinogenic’ to humans, respectively, but findings for bladder cancer remain inconsistent. **Objectives**: We assessed associations between processed meat and unprocessed red meat intake and bladder cancer risk using data from the Pooling Project of Prospective Studies of Diet and Cancer. **Methods**: Associations with bladder cancer risk were assessed using individual-level data from 30 cohort studies from North America, Europe, Australia and Asia. Diet was assessed at enrolment using validated food frequency questionnaires or diet histories. Following a pre-specified analysis plan, study- and sex-specific hazard ratios (HRs) and 95% confidence intervals (CIs) were estimated using Cox regression, adjusted for smoking status and duration and other potential confounders, and pooled using inverse-variance-weighted random-effects meta-analysis. **Results**: During median follow-up across cohorts of 8–29 years, among 2,575,971 participants, 20,780 were diagnosed with bladder cancer. Processed meat intake was associated with increased bladder cancer risk (HR comparing ≥40 with <5 g/day = 1.08; 95% CI: 1.02–1.15; HR per 20 g/day = 1.02; 95% CI: 1.00–1.03). Weaker evidence of association with increased risk was also observed for unprocessed red meat (HR comparing ≥100 with <20 g/day = 1.06; 95% CI: 1.00–1.13; HR per 50 g/day = 1.01; 95% CI: 0.99–1.03). There was no evidence of heterogeneity by study, sex, or region. **Conclusions**: Higher intakes of processed meat and unprocessed red meat were associated with a small increase in risk of bladder cancer. These findings are consistent with existing public health recommendations to limit consumption of red and processed meat.

## Simple Summary

Bladder cancer is one of the most common cancers worldwide, but there is still uncertainty about whether eating red and processed meat increases the risk of developing this disease. To provide clearer evidence, we analysed data from more than 2.5 million adults participating in 30 long-term studies conducted across North America, Europe, Australia and Asia. We examined whether people who consumed higher amounts of processed meat, such as bacon, ham and sausages, or unprocessed red meat, such as beef, lamb and pork, were more likely to be diagnosed with bladder cancer over time. We found that higher consumption of both processed meat and unprocessed red meat was associated with a small increase in bladder cancer risk, with stronger evidence for processed meat. These findings strengthen the evidence linking meat consumption to bladder cancer risk and are in line with public health recommendations to limit intake of red and processed meat.

## 1. Background

Bladder cancer is the 9th most common cancer globally and the 6th most common in males, with an estimated 613,791 new cases and 220,349 deaths in 2022 [1]. Most bladder cancers are urothelial carcinomas, while less common histological types include squamous cell carcinoma and adenocarcinoma [2]. Clinically, bladder cancer is generally classified as non-muscle-invasive or muscle-invasive disease, with approximately 70–75% of patients diagnosed with non-muscle-invasive bladder cancer [2,3]. Non-muscle-invasive bladder cancer is characterised by high recurrence rates, resulting in substantial treatment costs, making bladder cancer the most expensive cancer per patient to treat [4,5]. Bladder cancer risk is influenced by demographic, genetic and lifestyle factors, as well as environmental exposures [3,6,7]. Males have 3–5 times higher incidence than females [8]. Other established risk factors for bladder cancer include age, cigarette smoking, consuming water contaminated with arsenic or certain disinfection byproducts, and occupational exposure to bladder carcinogens, such as some arylamines, which are excreted in the urine, exposing the lining of the bladder to these agents [3,9].

Dietary factors may also influence bladder cancer risk due to the presence of carcinogens and their potentially carcinogenic metabolites, which are excreted through the urinary tract [10]. Identifying dietary factors associated with the risk of bladder cancer could inform prevention strategies [11].

Based on consistent evidence for colorectal cancer, the International Agency for Research on Cancer (IARC) has classified processed meat (of all types) as a Group 1 carcinogen (carcinogenic to humans) and unprocessed red meat as a Group 2A carcinogen (probably carcinogenic to humans) [12]. Meat-related carcinogens and their metabolites may be excreted in urine, where they can come into contact with the urothelium [13]. However, the evidence is more limited for bladder cancer. A recent meta-analysis of eight cohort studies and twelve case–control studies reported that higher intakes of processed meat and red meat were associated with 16%–23% increased risk, comparing highest and lowest categories. Stronger positive associations were observed in the case–control studies than in the cohort studies [14]. Further, findings from prospective studies included in the meta-analysis were inconsistent and limited by relatively small sample sizes, ranging from 114 to 1001 incident cases across eight cohorts [14]. In addition, the meta-analysis was subject to potential publication bias, as well as substantial heterogeneity across studies in dietary exposure assessment and adjustment for smoking and other confounders. The World Cancer Research Fund (WCRF) Continuous Update Project concluded that the evidence for associations between intakes of processed meat and unprocessed red meat with bladder cancer risk remains limited and inconclusive [15].

We aimed to address the limited available evidence on the associations between processed meat and unprocessed red meat consumption and bladder cancer risk by pooling harmonised individual-level data from 30 cohort studies from four continents, using standardised exposure categories and detailed adjustment for smoking status and duration.

## 2. Methods

### 2.1. Study Sample

The Pooling Project of Prospective Studies of Diet and Cancer (DCPP) is a consortium established to examine associations between dietary factors and cancer risk [16]. Thirty cohorts from North America, Europe, Australia and Asia were included in this pooled analysis (Table 1 and Supplementary Figure S1). The European Prospective Investigation into Cancer and Nutrition study (EPIC) included 8 European countries: Denmark, France, Germany, Italy, the Netherlands, Spain, Sweden, and the United Kingdom. Each included study met the following predefined criteria: (i) at least one publication on any diet and cancer association; (ii) ≥50 incident cases of bladder cancer for males or females; (iii) usual assessment of long-term diet sufficiently comprehensive to estimate total energy intake; (iv) validation of the dietary assessment tool or a closely related instrument [17,18,19,20,21,22,23,24,25,26,27,28,29,30,31,32,33,34,35,36,37,38,39,40,41,42,43,44,45,46]; and (v) assessment of processed meat or unprocessed red meat intake [16]. Participants in the included studies either provided written informed consent or were considered to have given informed consent through the completion of study questionnaires.

### 2.2. Dietary Assessment

Diet was assessed at baseline using self-administered food frequency questionnaires (FFQs), diet histories or interviewer-administered quantitative dietary questionnaires [16,27]. The dietary assessment tool used in each study, or a closely related instrument, had been evaluated in a validation study [17,18,19,20,21,22,23,24,25,26,27,28,29,30,31,32,33,34,35,36,37,38,39,40,41,42,43,44,45,46]. Most cohorts did not assess the validity of intakes of food groups [18,20,21,23,25,26,29,31,32,37,39,40,44,45,46], but cohort-specific correlations comparing the intake estimates from the FFQs versus multiple dietary records or 24-h recalls for total fat, saturated fat, total protein and cholesterol were generally greater than 0.40 [17,18,19,20,21,22,23,24,25,26,27,28,29,30,31,32,33,34,35,36,37,38,39,40,41,42,43,44,45,46]. Food intake data were converted to grams consumed per day using study-specific portion sizes and frequency of intake responses. Food group intakes were calculated by summing all related individual items within each cohort, with processed meat including sausages, hot dogs, bacon, ham and luncheon meats of any type (including poultry-based products), and unprocessed red meat including beef, pork, lamb and other red meat such as game meat and rabbit (but excluding organ meat). Total intakes of each of these two food groups were categorised using absolute cut-offs to maximise the exposure contrast while also ensuring sufficient incident case numbers across categories in each cohort: processed meat <5, 5 to <10, 10 to <20, 20 to <40 and ≥40 g/day and unprocessed red meat <20, 20 to <40, 40 to <60, 60 to <100 and ≥100 g/day.

Processed meat intake was generally very low for participants in the Shanghai Women’s Health Study (SWHS), so this study was excluded from the processed meat analyses.

### 2.3. Ascertainment of Cases

Incident cases of first primary bladder cancer were defined using *International Classification of Diseases*, Ninth Revision (ICD-9) codes 188.0–188.9 or 233.7, or *International Classification of Diseases*, Tenth Revision (ICD-10) codes C67.0–C67.9, and ascertained by record linkage to cancer and death registries or self-report confirmed by review of medical records [16]. Cases with behaviour codes 2 (Tis, Ta) or 3 (T1–T4) were included. Cases with unknown or missing histology were included, while those with behaviour code 0 (benign), 1 (uncertain), 6 (metastatic, not originating in the bladder), or 9 (unknown) were censored at diagnosis and not classified as bladder cancer cases.

### 2.4. Participant-Level Exclusions

In addition to study-specific exclusions that were applied by individual cohorts, participants were excluded from the analyses if they met any of the following criteria: (i) personal history of cancer (except for non-melanoma skin cancer) at baseline; (ii) total energy intake beyond three standard deviations from their study- and sex-specific log_e_-transformed mean energy intake; (iii) missing data on smoking status; (iv) missing data for the exposure of interest.

### 2.5. Statistical Analysis

We analysed individual participant-level data separately by cohort and by sex. The Netherlands Cohort Study (NLCS) and the Canadian Study of Diet, Lifestyle and Health (CSDLH) were analysed as case–cohort studies [16,47].

The primary analysis was conducted in two stages. In stage 1, study- and sex-specific hazard ratios and 95% confidence intervals were calculated using Cox regression [48]. We stratified jointly by age at baseline (years), the year in which the baseline questionnaire was returned, and country (for EPIC only), treating follow-up time since study entry as the time scale (until the date of any incident cancer diagnosis, death, loss to follow-up, or administrative end of follow-up, whichever came first). In stage 2, random-effects meta-analysis was used to pool study- and sex-specific hazard ratios, which were weighted by the inverse of the sum of their variance and the estimated between-studies variance component [49]. Heterogeneity of associations across studies was evaluated by I^2^ and Q statistics [49,50,51]. The assumption of proportional hazards was assessed for each sex-specific cohort based on scaled Schoenfeld residuals [52], with *p* < 0.001 considered evidence of departure for a given cohort and exposure, given the hundreds of tests conducted.

Epidemiological studies have consistently demonstrated that bladder cancer risk is strongly associated with smoking history, with smoking status and duration being key determinants of risk [3]. Therefore, in the primary analyses, smoking habits were adjusted for by including smoking status and duration in multivariable models. For the Swedish Mammography Cohort (SMC), 1997 was considered baseline because smoking habits were first assessed in that year.

A causal diagram was used to determine which potentially confounding variables to include in stage 1 multivariable models (Supplementary Figure S2). Multivariable models included the following covariates: self-identified race and ethnicity (Asian, Black, Hispanic, White, other), educational level (<high school, high school, >high school), smoking status and duration (never smoker, former smoker < 30 years duration, former smoker ≥ 30 years duration, current smoker < 45 years duration, current smoker ≥ 45 years duration), alcohol consumption (continuous, g/day), physical activity (low, medium, high), total fruit (including fruit juice) intake (continuous, g/day), total vegetable (including vegetable juice) intake (continuous, g/day) and total energy intake (continuous, kcal/day). Participants from cohorts without smoking duration data (NIH-AARP, CLUE II, JPHC I, and JPHC II) were categorised based on smoking status only. Models assessing processed meat intake were additionally adjusted for unprocessed red meat intake (continuous, g/day), and vice versa. For studies with at least 200 incident cases, we adjusted for covariates directly in the model; for studies with less than 200 cases, we applied propensity scores to reduce overfitting [53,54]. For continuous exposures, propensity scores were calculated using linear regression of the exposure on the same covariates in the multivariable model, while for categorical exposures, a logistic regression model was used to estimate the probability of each exposure category. The predicted values or probabilities were included as adjustment variables in the Cox model together with the exposure. The missing indicator method was used for covariates with missing data.

To assess linearity of associations between intake of each type of meat and risk of bladder cancer, we compared models with a linear term to the corresponding models with additional cubic spline terms with 4 knots placed at the 5th, 35th, 65th, and 95th percentiles using the likelihood ratio test, based on the aggregated analysis method in which individual-level data were combined and analysed together with study and sex as additional stratifying variables; participants with intake above the 99th percentile, calculated by pooling consumption data across all cohorts, were excluded.

Trend tests were conducted by including the median value of each exposure category as a continuous variable in the models and evaluating the *p*-value for trend, calculated based on the Wald test.

Effect modification by sex, smoking status (never, former, current), geographic region (North America, Asia, Europe/Australia), attained age (<70, 70–74.9 and ≥75 years), and duration of follow-up (<5, 5–9.9, and ≥10 years) was assessed via meta-regression of study-specific results [55]. Effect modification by race (Asian, Black, White) was assessed using aggregated analysis.

To evaluate potential reverse causation, we conducted sensitivity analyses examining associations between processed meat and unprocessed red meat consumption and bladder cancer risk after excluding the first two years of follow-up. We performed another sensitivity analysis restricted to the 21 cohorts in which non-invasive papillary carcinoma (Ta) and carcinoma in situ (Tis) were systematically ascertained, as confirmed by the principal investigators of each cohort, to reduce heterogeneity in the definition of cases.

To evaluate the potential confounding effects of smoking on the associations between intake of processed meat and unprocessed red meat and bladder cancer risk, we performed two sensitivity analyses. In the first, we restricted analyses to cohorts with complete data on smoking status and duration (excluding CLUE II, NIH-AARP, JPHC I, and JPHC II) and compared hazard ratios from models with minimal adjustments (stratified only on baseline age, questionnaire completion year, and country [EPIC]) to models additionally adjusted for: (i) smoking status (never, former, or current smoker) and (ii) combined smoking status and duration (never smoker, former smoker with less than 30 years duration, former smoker with at least 30 years duration, current smoker with less than 45 years duration, current smoker with at least 45 years duration). Additionally, we compared these results with models adjusting for all covariates except smoking, progressively incorporating smoking status and duration. In the second sensitivity analysis, we included only participants with complete data on smoking status, duration, intensity (number of cigarettes smoked per day, available for current smokers only), and time since cessation for former smokers, and only studies with <10% missing data across all of these smoking-related variables. We compared models including all covariates except smoking with those sequentially adding smoking status, duration, intensity, and time since quitting. To evaluate potential residual confounding from occupational exposures, we conducted two sensitivity analyses. The first was restricted to participants with more than a high school education. The second was limited to cohorts of teachers and health professionals, including the California Teachers Study (CTS), Nurses’ Health Study (NHS) and NHS II (all female-only), and the Health Professionals Follow-up Study (HPFS; male-only; ClinicalTrials.gov Registration ID: NCT00005182; specific registration page: https://clinicaltrials.gov/study/NCT00005182 (accessed on 1 September 2026)). Each cohort and the consortium received approval from its institutional review board, and participants either provided informed consent directly or informed consent was implied through completion of study questionnaires by participants. All analyses were conducted using SAS software version 9.4 (SAS Institute, Cary, NC, USA).

## 3. Results

Across 30 cohort studies with a median follow-up of 8–29 years, analyses of unprocessed red meat included 2,575,971 participants (1,538,168 female and 1,037,803 male), of whom 20,780 (5297 female and 15,483 male) were diagnosed with incident bladder cancer; the corresponding numbers for analyses of processed meat (excluding SWHS) were 2,502,700 cohort study participants (1,465,201 female and 1,037,499 male) and 20,672 incident cases (5199 female and 15,473 male) (Table 1). There was considerable variation across studies in processed meat and unprocessed red meat intake, with differences in the study-specific median intakes ranging from 6-fold for unprocessed red meat (CTS: 16.0 g/day vs. Canadian National Breast Screening Study [CNBSS]: 91.0 g/day, Supplementary Figure S4A,B) to 25-fold for processed meat (Singapore Chinese Health Study [SCHS]: 1.4 g/day vs. EPIC: 36.0 g/day, Supplementary Figure S3A,B).

**Figure 1:**
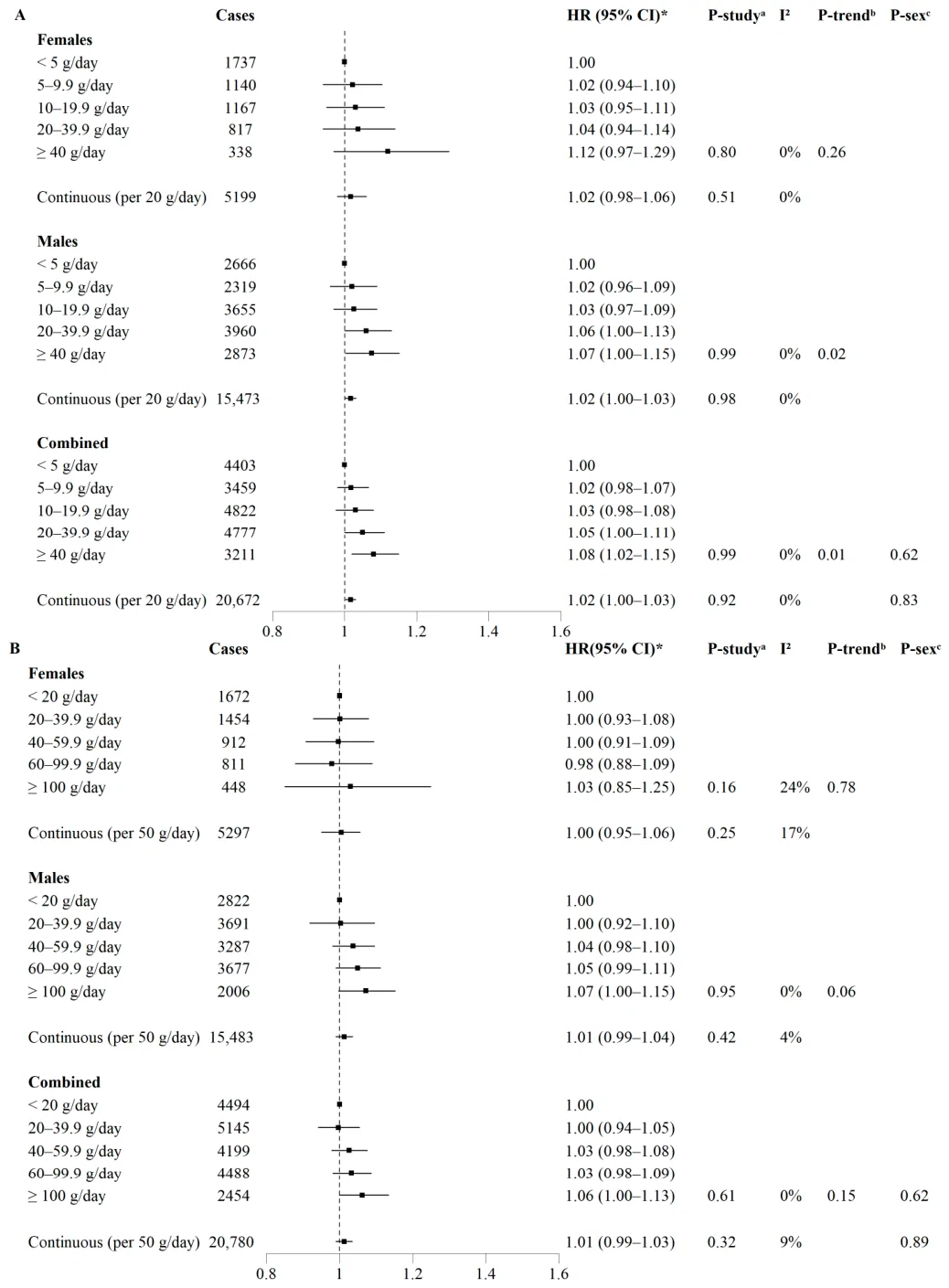
Associations between processed meat (**A**) and unprocessed red meat (**B**) consumption and risk of bladder cancer in females, males and all participants. HR, hazard ratio; CI, confidence interval. * All models were stratified jointly on baseline age (in years), year of baseline questionnaire completion, and centre (EPIC only) and adjusted for race and ethnicity, smoking status and duration, educational level, alcohol consumption, physical activity, total fruit (including fruit juice) intake, total vegetable (including vegetable juice) intake, and total energy intake; analyses for processed meat were additionally adjusted for unprocessed red meat and vice versa; these variables were entered directly into the multivariable model or, for studies with less than 200 cases, modelled using propensity scores. ^a^ Between-study heterogeneity was tested using the Q-statistic. ^b^ Linear trend was tested using a continuous variable based on the median of each category. ^c^ Heterogeneity by sex was examined using meta-regression.

In multivariable analyses, consuming ≥40 g/day of processed meat was associated with an estimated 8% increased risk of bladder cancer overall (HR = 1.08; 95% CI: 1.02–1.15), relative to consuming <5 g/day (Figure 1A). Compared with minimally adjusted models, these estimates reflect substantial attenuation after adjustment, indicating confounding by the covariates considered (Supplementary Figure S5A). There was no evidence of heterogeneity by study (*p* = 0.99) or by sex (*p* = 0.62) for the comparisons of highest and lowest categories (Figure 1A and Supplementary Figure S6A,B).

In multivariable analyses, consuming ≥100 g/day of unprocessed red meat was associated with an estimated 6% increased risk of bladder cancer compared to consuming <20 g/day (HR 1.06; 95% CI: 1.00–1.13, Figure 1B). Compared with minimally adjusted models (Supplementary Figure S5B), this and other HR estimates were also substantially attenuated. There was no evidence of heterogeneity by study (*p* = 0.61) or by sex (*p* = 0.62; Figure 1B and Supplementary Figure S7A,B).

There was no evidence of departure from linearity, overall or by sex, for the associations between either processed meat or unprocessed red meat consumption and bladder cancer risk (*p* ≥ 0.23). When processed meat intake was modelled linearly in multivariable-adjusted models, the estimated HR per 20 g/day increment was 1.02 (95% CI: 1.00–1.03; *p* = 0.01) (Figure 1A). For unprocessed red meat, the estimated HR per 50 g/day increment was 1.01 (95% CI: 0.99–1.03; *p* = 0.15; Figure 1B).

**Figure 2:**
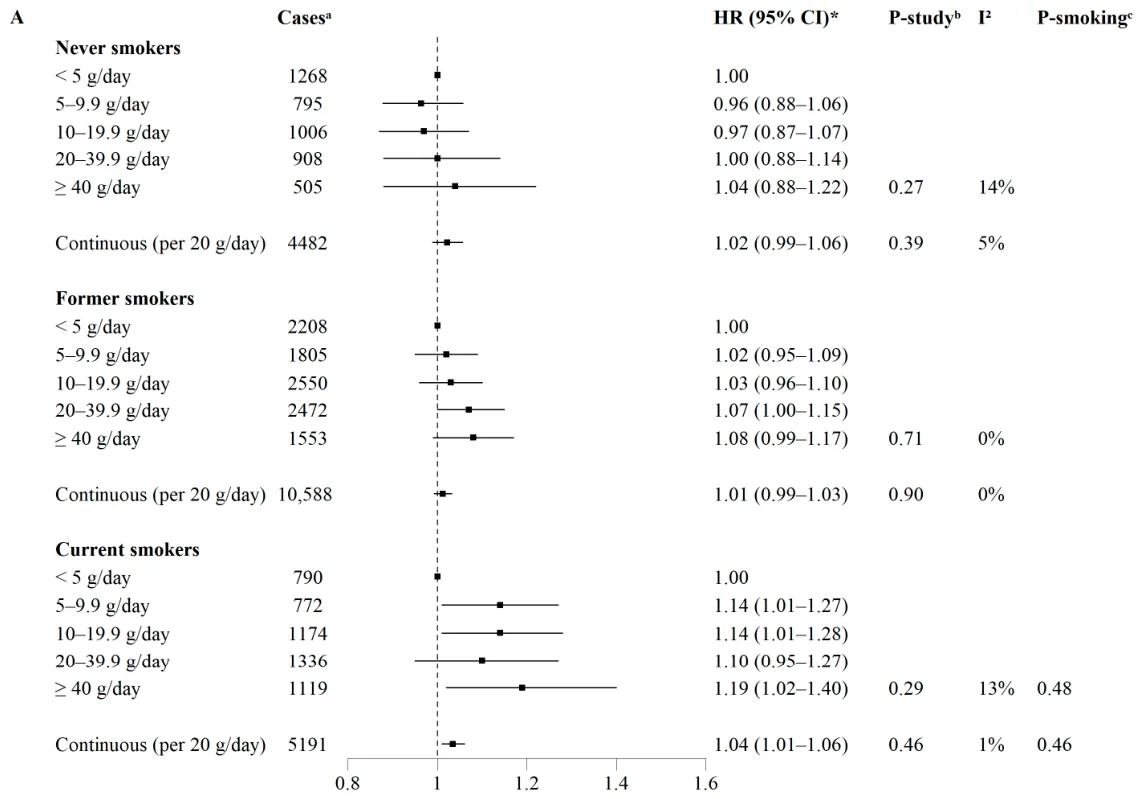

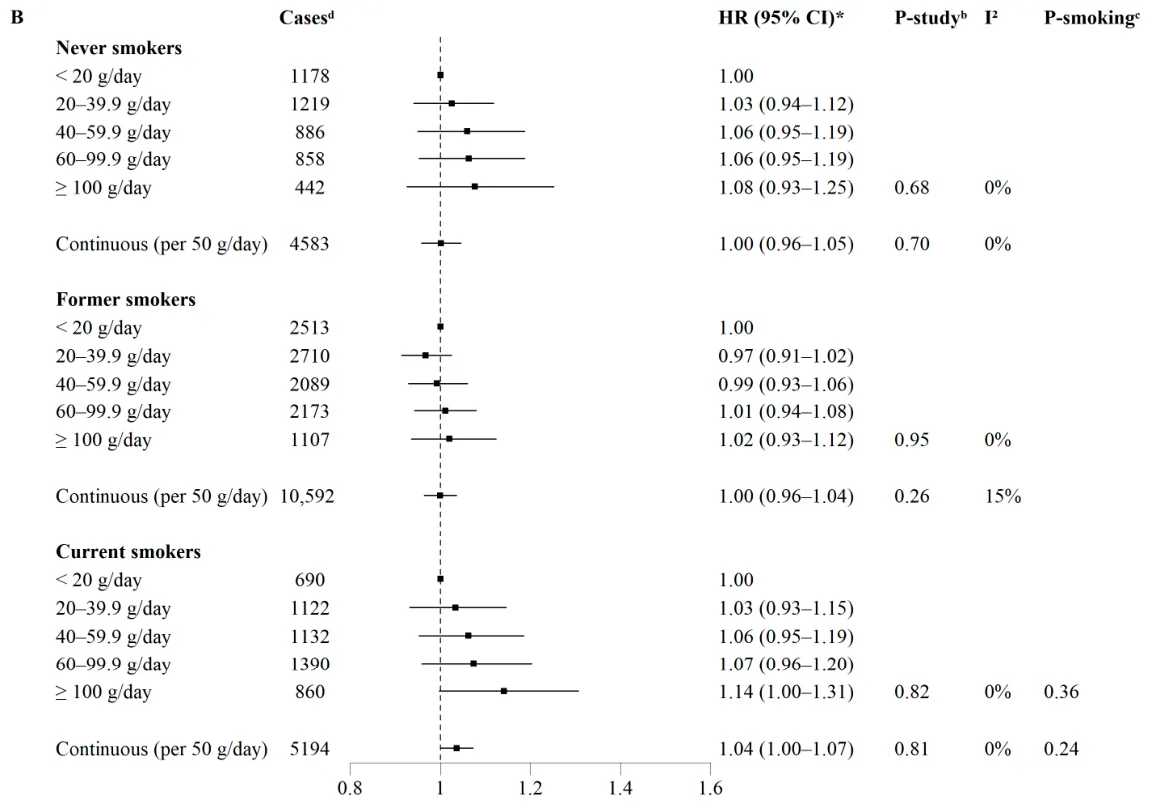
Association between processed meat (**A**) and unprocessed red meat (**B**) consumption and risk of bladder cancer by smoking status. HR, hazard ratio; CI, confidence interval. * All models were stratified jointly on baseline age (in years), year of baseline questionnaire completion, and centre (EPIC only) and adjusted for race and ethnicity, smoking duration for former and current smokers, educational level, alcohol consumption, and physical activity; analyses for processed meat were additionally adjusted for unprocessed red meat and vice versa, total fruit (including fruit juice) intake, total vegetable (including vegetable juice) intake, and total energy intake; these variables were entered directly into the multivariable model or, for studies with less than 200 cases, modelled using propensity scores. ^a^ For processed meat, BWHS, CARET, CLUE II, JPHC I, NYSC (females only), SCCS, and WLHS were excluded from the never-smoker analysis; JPHC I was excluded from the former smokers analysis for males; and BWHS, CARET, NHS II, NYSC, SCCS and WLHS were excluded from the former smokers analysis for females, in each case because there were fewer than 25 cases; SMC was excluded from female analysis due to 0 cases in the reference category. For current smokers, CLUE II and CSDLH were excluded from the male analysis, and BCDDP, BWHS, MCCS, NHS II, NYSC, PLCO, and VITAL were excluded from the female analysis, all due to small case numbers (<25). ^b^ Between-study heterogeneity was tested using the Q-statistic. ^c^ Heterogeneity by smoking status was tested using meta-regression. ^d^ For red meat, BWHS, CARET, CLUE II, JPHC I, NYSC (females only), SCCS, and WLHS were excluded from the never-smoker analysis; JPHC I was excluded from the former smokers analysis for males; and BWHS, CARET, NHS II, NYSC, SCCS, SWHS, and WLHS were excluded from the former smokers analysis for females, in each case because there were fewer than 25 cases. For current smokers, CLUE II and CSDLH were excluded from the male analysis, and BCDDP, BWHS, MCCS, NHS II, NYSC, PLCO, SWHS, and VITAL were excluded from the female analysis, all due to small case numbers (<25). *p*-values for heterogeneity by sex for processed meat intake among never smokers, former smokers and current smokers were 0.39, 0.82 and 0.40, respectively, for the continuous term and 0.07, 0.71 and 0.57, respectively, for the comparison of the highest versus lowest consumption category. *p*-values for heterogeneity by sex for unprocessed red meat intake among never smokers, former smokers and current smokers were 0.27, 0.66 and 0.77, respectively, for the continuous term and 0.13, 0.82 and 0.97, respectively, for the comparison of the highest versus lowest consumption category.

There was no statistical evidence of heterogeneity in associations with bladder cancer risk by smoking status for the highest versus lowest categories of intake or when intake was modelled continuously for processed meat (*p* ≥ 0.46) or unprocessed red meat (*p* ≥ 0.24), although HR estimates were substantially higher for current smokers. Among current smokers, the HRs comparing the highest versus lowest categories were 1.19 (95% CI: 1.02–1.40, Figure 2A) for processed meat and 1.14 (95% CI: 1.00–1.31, Figure 2B) for unprocessed red meat, and the HRs per 20 g/day and 50 g/day increment were 1.04 (95% CI: 1.01–1.06) and 1.04 (95% CI: 1.00–1.07), respectively.

Results from analyses restricted to the 25 cohorts that had assessed smoking status and duration suggested that, compared with minimally adjusted models, adjusting for smoking status (never, former, current) substantially attenuated associations of processed meat and unprocessed red meat intakes with bladder cancer risk (Supplementary Table S5). Further adjustment for smoking duration had a limited effect on HR estimates, as did adjusting for covariates other than those related to smoking. Additional sensitivity analyses restricted to participants with complete data on smoking status, duration, intensity, and time since cessation, from studies with <10% missing data on any of these covariates, showed that further adjustment for smoking intensity and time since cessation also had minimal effect on estimates (Supplementary Table S6).

We observed no evidence of heterogeneity in associations by geographic region or race for processed meat or unprocessed red meat (*p* ≥ 0.15; Supplementary Tables S1 and S2), although the estimated association for processed meat intake was strongest among Black participants (HR = 1.51; 95% CI: 1.10–2.08). For processed meat intake, we found some evidence of heterogeneity by follow-up time (*p* = 0.03), but no clear trend was observed across the follow-up time strata (e.g., for the highest vs. lowest intake category comparison, less than 5 years: HR 1.08; 95% CI: 0.95–1.22; 5–9.9 years: HR 1.24; 95% CI: 1.09–1.41; 10 years or more: HR 1.01; 95% CI: 0.92–1.11; Supplementary Table S3). For unprocessed red meat intake, we found some evidence of heterogeneity by attained age: the HR comparing ≥100 g/day with <20 g/day was 1.15 (95% CI: 1.04–1.27) for participants aged less than 70 years, 1.14 (95% CI: 0.95–1.35) for participants aged between 70 and 75 years, and 0.97 (95% CI: 0.85–1.11) for those aged 75 years or older (*p* = 0.08; Supplementary Table S4).

Results were consistent for both processed meat and unprocessed red meat when we excluded the first 2 years of follow-up from the analyses (Supplementary Figure S8), after restricting analyses to the 21 studies that systematically included Tis and Ta bladder cancer cases (Supplementary Figure S9), and after excluding data from sex-specific cohorts for which evidence of departure from proportional hazards was observed at *p* < 0.001 (Cancer Prevention Study [CPS II] males for red meat and Multiethnic Cohort Study [MEC] females for both meat groups; Supplementary Figure S10). Results were also consistent when analyses were restricted to cohorts of teachers or health professionals and among participants with more than a high school education (Supplementary Table S7).

## 4. Discussion

In this pooled analysis of over 2.5 million participants in 30 prospective cohort studies, including more than 20,000 incident cases, we found that higher intakes of processed meat and unprocessed red meat were associated with small increases in risk of bladder cancer. Results were generally consistent across studies and strata defined by sex and geographical region, with much wider confidence intervals for females due to the substantially smaller number of cases.

Our findings extend the evidence from prior research on meat consumption and bladder cancer risk. A recent meta-analysis estimated a relative risk for bladder cancer comparing the highest versus lowest processed meat intake categories of 1.16 (95% CI: 1.08–1.25) [14]. When the meta-analysis was limited to cohort studies, five [23,28,33,35,38] of which were also included in our pooled analyses, the association was weaker (relative risk (RR) 1.08; 95% CI: 0.95–1.22) and similar to our findings in the 30 cohort studies included here. Our study provides a more precise estimate, based on categories of exposure that were consistent across cohorts. Approximately 7% of females and 19% of males reported consuming more than 40 g of processed meat per day, which is roughly equivalent to one hot dog, a small sausage or two slices of bacon per day.

For unprocessed red meat, previous meta-analyses have consistently reported stronger associations in case–control studies and weak evidence of associations in cohort studies [14,56]. A meta-analysis conducted in 2018 yielded a modest positive dose–response relationship between red meat intake and bladder cancer overall (RR per 100 g/day = 1.27; 95% CI: 1.03–1.57), whereas little evidence of association was observed across cohort studies (RR per 100 g/day = 0.95; 95% CI: 0.82–1.11), up to six of which [23,27,28,33,35,41] were included in our pooled analyses [56].

For both processed meat and unprocessed red meat, the stronger associations observed in case–control studies might be explained by recall and selection bias [57]. In contrast, our findings based on pooled prospective data from 30 cohorts suggest there is a weak, positive association, which likely provides a more valid estimate of the true association. While previous meta-analyses also included cohorts from multiple regions, our harmonised individual-level data from 30 cohorts allowed a more consistent assessment of effect modification by race or geographic region.

Smoking is one of the most important risk factors for bladder cancer [3]. Experimental studies suggest that cigarette smoking may amplify the carcinogenic potential of meat, possibly exerting a synergistic effect on cancer risk [58,59]. More specifically, a higher total intake of N-nitroso compounds (NOCs) may elevate bladder cancer risk among meat consumers who are current smokers, given the presence of NOCs in meat and the formation of tobacco-specific nitrosamines from cigarette smoking [60]. Consistent with this, we observed stronger associations in current smokers than in never and former smokers, although in this very large sample there was little statistical evidence of heterogeneity in associations by smoking status [61,62].

Our pooled analysis found that associations for processed meat consumption differed by follow-up time, but there was no clear pattern with increasing time. We also found some evidence that intake of unprocessed red meat was associated with increased bladder cancer risk before age 75 years and little evidence of association after age 75 years. The decline in immune surveillance with aging contributes to an increased overall cancer risk [63]. It is also possible that individuals remaining cancer-free after age 75 are less susceptible to cancer [64]. This selection may attenuate the associations between dietary intake and bladder cancer risk in older age groups.

Notably, the estimated association between processed meat intake and bladder cancer risk was strongest in Black participants. In our pooled analyses, 16.9% of Black participants, 16.3% of White participants and 4.8% of Asian participants were in the highest category for processed meat intake. At the upper end of the distribution (95th and 99th percentiles), Black individuals reported higher processed meat consumption (78.2 and 148.8 g/day, respectively), compared with White individuals (64.2 and 112.9 g/day) and Asian individuals (30.4 and 57.4 g/day), which could partly explain the stronger association observed. Although the test for heterogeneity by race was not statistically significant, the small number of Black cases may have limited the power to detect interaction.

There are several biological mechanisms that could explain associations between intakes of processed meat and unprocessed red meat and risk of bladder cancer. One proposed mechanism involves the formation of chemical carcinogens during meat cooking and processing [12]. Processed meat and unprocessed red meat cooked at high temperatures contain pro-carcinogenic compounds such as heterocyclic amines and polycyclic aromatic hydrocarbons. Some heterocyclic amines have been shown to induce urinary bladder tumours in experimental animals, and human bladder epithelial cells can bioactivate certain heterocyclic aromatic amines to DNA-damaging metabolites [13,65]. Nitrates and nitrites, added to processed meat for preservation, colour and flavour enhancement, are precursors to NOCs [66]. Additionally, both unprocessed and processed red meats contain heme iron, which increases the endogenous synthesis of NOCs [67,68]. Experimental studies have also shown that some N-nitroso compounds can induce mutagenesis in the urothelium and cause bladder tumours [69]. NOCs are potentially carcinogenic compounds known to enhance tumour growth [70,71].

This pooled analysis has several strengths. First, it combined data from 30 cohort studies across four continents, with more than 20,000 incident bladder cancer cases. The large sample size provided much greater statistical power to examine associations than individual studies and allowed us to examine whether associations varied by population subgroups, although power remained limited to detect modest differences in some subgroup analyses, particularly those by race. Second, the prospective design minimised the effects of recall bias and selection bias, which are common in case–control studies [57]. Third, the variation in meat consumption across cohorts allowed us to assess associations over the full range of exposure. We also harmonised exposure, covariate, and outcome definitions across studies, which helped reduce heterogeneity and ensured more consistent comparisons. In addition, our findings are less likely to be influenced by publication bias, as 20 of the 30 cohort studies in our pooled analyses, contributing 7530 bladder cancer cases, had not previously published on associations between meat consumption and bladder cancer risk. Finally, the comprehensive baseline data from DCPP, including detailed demographic and smoking information, allowed us to apply the most comprehensive smoking adjustment models and control for other potential confounders. We conducted sensitivity analyses that incorporated more detailed smoking information, including smoking intensity and time since cessation, in addition to smoking status and duration. Results were consistent with those from the main analyses, suggesting that residual confounding by smoking was minimal, although it cannot be entirely ruled out.

Our study has some limitations. First, we were unable to account for occupational exposure to carcinogenic chemicals, which is an established risk factor for bladder cancer. However, in our sensitivity analyses limited to cohorts of teachers, nurses and health professionals (which are likely low-risk occupations) or restricted to participants with education more than high school, the associations were generally consistent, indicating that our results are unlikely to be explained by occupational confounding. Although we adjusted for various lifestyle-related risk factors, we cannot completely rule out the possibility that our findings may partly reflect associations with unmeasured lifestyle factors associated with meat intake [72]. Another limitation is that dietary intake was assessed primarily using food frequency questionnaires, which are subject to measurement error and potential misclassification [73]. Biomarker data on meat intake were not available in the pooled dataset used for this analysis. Given that median follow-up ranged from 8 to 29 years across cohorts, our baseline dietary assessment may not fully represent long-term intake or changes in dietary and lifestyle factors during follow-up. However, given the relatively long latency of bladder cancer, baseline dietary intake may still capture an etiologically relevant exposure period. Non-differential measurement error from a single baseline dietary assessment may have attenuated the observed associations towards the null. Further, we were unable to capture variation in meat preparation and cooking methods across cohorts or assess potential differences in composition of processed meats. Factors such as trimming fat, use of lean versus fatty cuts, cooking temperature, and cooking methods like roasting, grilling or stewing may influence the production of carcinogens [74].

## 5. Conclusions

In summary, our pooled analysis found small positive associations between intakes of processed and unprocessed red meat and bladder cancer risk. Our findings are consistent with existing public health recommendations to limit consumption of red and processed meat.

## Figures and Tables

**Table 1:** Characteristics of the cohort studies in the pooled analysis of unprocessed red meat and bladder cancer risk. (**A**) Cohort characteristics, meat intake, and smoking status. (**B**) Participants excluded and missing covariate data.

(**A**)													
**Study †**	**Country/Continent**	**Sex**	**Baseline Cohort** **Size ‡**	**Total Cases**	**Age at Baseline** **(Years) §**		**Year of Baseline** **Questionnaire**	**Follow-Up Time** **(Years) §**	**Meat Intake, Median (IQR), g/Day**		**Smoking Status (%)**		
									**Unprocessed** **Red Meat**	**Processed** **Meat**	**Never**	**Past**	**Current**
BCDDP	USA	female	41,754	87	60 (51–72)		1987–1989	9 (9–10)	24 (12–41)	6 (2–15)	57	31	12
BWHS	USA	female	50,743	66	37 (25–53)		1995	25 (16–25)	18 (8–36)	8 (2–18)	65	20	15
CARET	USA	male	10,474	333	57 (50–66)		1985–1994	13 (5–16)	50 (31–79)	16 (8–31)	1	43	56
CARET	USA	female	6000	64	57 (51–66)		1985–1994	13 (6–16)	29 (17–47)	7 (2–13)	0	32	68
CLUE II	USA	male	6229	95	48 (29–69)		1989	29 (8–29)	44 (27–70)	22 (11–39)	43	40	17
CNBSS	Canada	female	48,788	190	48 (41–57)		1980–1985	22 (17–24)	91 (64–124)	14 (7–23)	51	29	20
COSM	Sweden	male	43,963	765	59 (48–74)		1998	19 (5–19)	57 (38–80)	33 (20–47)	36	39	25
CPS II	USA	male	65,465	1489	64 (56–72)		1992–1993	14 (4–25)	44 (26–70)	13 (5–27)	33	58	9
CPS II	USA	female	73,726	399	62 (54–71)		1992–1993	18 (5–25)	26 (14–43)	6 (1–13)	55	36	9
CSDLH	Canada	male	34,291	104	70 (56–83)		1991–1999	11 (3–16)	37 (23–54)	19 (9–36)	40	56	4
CTS	USA	female	92,243	350	50 (34–71)		1995–1999	24 (6–24)	16 (6–29)	5 (1–13)	68	27	5
EPIC	Europe	male	129,580	1324	52 (40–63)		1991–2000	16 (8–18)	47 (22–80)	36 (19–60)	34	37	29
EPIC	Europe	female	278,507	521	51 (37–63)		1991–2000	15 (8–18)	34 (13–59)	21 (10–38)	58	23	18
HPFS	USA	male	45,866	835	54 (42–68)		1986–1987	26 (7–31)	56 (30–98)	7 (2–13)	46	44	10
IWHS	USA	female	34,108	235	61 (56–68)		1986	22 (6–28)	80 (44–109)	5 (1–9)	66	19	15
JPHC I	Japan	male	18,186	99	55 (46–63)		1995	18 (7–18)	37 (21–65)	4 (2–11)	36	17	47
JPHC II	Japan	male	21,549	119	58 (47–70)		1998–1999	15 (5–15)	33 (18–58)	3 (1–7)	35	19	46
MCCS	Australia	male	14,456	412	55 (42–66)		1990–1994	21 (8–23)	70 (46–104)	21 (10–36)	43	42	15
MCCS	Australia	female	21,918	125	54 (42–66)		1990–1994	21 (11–23)	49 (30–72)	13 (6–23)	69	22	9
MEC	USA	male	80,372	1476	60 (47–72)		1993–1998	21 (5–27)	39 (21–65)	15 (7–27)	30	52	18
MEC	USA	female	89,847	490	59 (47–72)		1993–1998	24 (7–27)	26 (14–45)	9 (4–18)	56	29	14
NHS	USA	female	68,249	381	53 (43–63)		1986–1987	28 (10–29)	48 (33–85)	7 (3–10)	44	35	21
NHS II	USA	female	92,933	57	36 (30–42)		1991–1993	22 (21–22)	55 (35–85)	6 (2–8)	66	22	12
NIH-AARP	USA	male	283,685	5332	62 (54–68)		1995–1997	16 (3–16)	39 (20–68)	17 (8–33)	30	59	11
NIH-AARP	USA	female	192,347	986	62 (53–68)		1995–1997	16 (4–16)	23 (11–43)	8 (4–17)	46	40	15
NLCS	Netherlands	male	58,279	1526	62 (56–67)		1986	14 (4–17)	60 (45–78)	18 (9–30)	11	50	39
NLCS	Netherlands	female	62,573	270	61 (56–68)		1986	17 (8–17)	52 (36–69)	12 (6–21)	57	21	22
NYSC	USA	male	28,751	199	60 (47–74)		1980	8 (4–8)	60 (52–60)	15 (7–27)	29	50	21
NYSC	USA	female	21,668	57	65 (47–80)		1980	8 (5–8)	54 (12–60)	10 (4–18)	50	27	23
PLCO	USA	male	49,424	568	65 (59–74)		1997–2004	9 (4–11)	51 (29–87)	15 (8–28)	38	52	10
PLCO	USA	female	52,220	125	65 (58–73)		1997–2004	9 (6–11)	32 (17–55)	7 (4–14)	58	34	8
SCCS	USA	male	29,054	134	50 (42–63)		2002–2009	11 (4–15)	78 (40–147)	33 (14–62)	24	24	52
SCCS	USA	female	41,991	60	51 (42–65)		2002–2009	12 (7–15)	48 (23–94)	14 (5–34)	46	21	33
SCHS	Singapore	male	27,176	132	56 (46–68)		1993–1999	14 (6–17)	26 (16–42)	1 (0–4)	42	21	37
SMC	Sweden	female	33,768	138	60 (51–75)		1997	19 (7–19)	29 (14–44)	21 (12–33)	54	23	23
SMHS	China	male	61,136	209	53 (43–70)		2001–2006	12 (9–14)	49 (31–73)	2 (0–6)	30	11	59
SWHS ^a^	China	female	72,848	98	50 (42–66)		1996–2000	18 (13–19)	42 (25–63)	—	97	1	2
VITAL	USA	male	29,867	332	60 (52–72)		2000–2002	10 (5–11)	59 (28–100)	15 (6–30)	40	51	9
VITAL	USA	female	30,065	85	59 (52–72)		2000–2002	10 (7–11)	35 (16–64)	5 (2–13)	57	36	7
WHI	USA	female	84,483	454	63 (53–73)		1992–1995	22 (7–25)	26 (12–50)	6 (2–14)	51	42	7
WLHS	Sweden	female	47,389	59	40 (32–48)		1991–1992	21 (20–21)	46 (30–63)	4 (2–7)	41	37	22
Total			2,575,971	20,780									
(**B**)													
**Study †**	**Sex**	**Participants Excluded, *n* ‖**			**Missing Covariate Data (%) #**								
					**Race/Ethnicity**	**Education**		**Physical Activity**			**Alcohol Intake**		
BCDDP	female	390			0	0.8		12.2			0		
BWHS	female	56			0	0.2		0.3			0		
CARET	male	0			<0.1	11.0		100			0		
CARET	female	0			<0.1	7.3		100			0		
CLUE II	male	0			<0.1	0		100			0		
CNBSS	female	0			0	1.3		15.4			0		
COSM	male	1257			0	0.7		6.8			0		
CPS II	male	431			0.3	0.6		1.0			0		
CPS II	female	878			0.2	0.6		0.9			0		
CSDLH	male	6			<0.1	<0.1		<0.1			0		
CTS	female	545			0	0		0.4			0		
EPIC	male	1377			0	2.4		1.8			0		
EPIC	female	5123			0	4.3		1.1			0		
HPFS	male	1902			1.7	0		0.4			0		
IWHS	female	470			1.0	0.2		1.4			0		
JPHC I	male	228			0	19.1		0			0		
JPHC II	male	2118			0	100		0			0		
MCCS	male	1			0	0		0			0		
MCCS	female	0			0	0		0			0		
MEC	male	989			7.1	0.4		2.8			0		
MEC	female	1800			0	0.6		4.5			0		
NHS	female	193			6.4	0		0.5			0		
NHS II	female	155			1.5	0		0.4			0		
NIH-AARP	male	11,617			1.0	2.5		0.7			0		
NIH-AARP	female	7125			1.4	3.1		1.1			0		
NLCS	male	0			0	<0.1		<0.1			0		
NLCS	female	0			0	<0.1		<0.1			0		
NYSC	male	1585			0.3	3.4		100			0		
NYSC	female	867			0.7	6.8		100			0		
PLCO	male	10			<0.1	0.2		52.0			0		
PLCO	female	3			<0.1	0.2		55.0			0		
SCCS	male	244			0.5	0.5		1.3			0		
SCCS	female	205			0.3	0.3		1.2			0		
SCHS	male	0			0	0		0			0		
SMC	female	1214			0	0.2		4.9			0		
SMHS	male	0			0	0.2		0			0		
SWHS ^a^	female	0			0	<0.1		0			0		
VITAL	male	303			0.4	0.2		1.3			0		
VITAL	female	91			0.4	0.5		1.2			0		
WHI	female	1065			0.2	0.8		1.2			0		
WLHS	female	149			0	16.6		4.4			0		
Total		42,397											

† Study abbreviations: BCDDP: Breast Cancer Detection Demonstration Project Follow-Up Study; BWHS: Black Women’s Health Study; CARET: Beta-Carotene and Retinol Efficacy Trial; CLUE II: Campaign against Cancer and Heart Disease; CNBSS: Canadian National Breast Screening Study; COSM: Cohort of Swedish Men; CPS II: Cancer Prevention Study II Nutrition Cohort; CSDLH: Canadian Study of Diet, Lifestyle and Health; CTS: California Teachers Study; EPIC: European Prospective Investigation into Cancer and Nutrition; HPFS: Health Professionals Follow-up Study; IWHS: Iowa Women’s Health Study; JPHC I: Japan Public Health Center-based Prospective Study I; JPHC II: Japan Public Health Center-based Prospective Study II; MCCS: Melbourne Collaborative Cohort Study; MEC: Multiethnic Cohort Study; NHS: Nurses’ Health Study; NHS II: Nurses’ Health Study II; NIH-AARP: NIH-AARP Diet and Health Study; NLCS: Netherlands Cohort Study; NYSC: New York State Cohort; PLCO: Prostate, Lung, Colorectal, and Ovarian Cancer Screening Trial; SCCS: Southern Community Cohort Study; SCHS: Singapore Chinese Health Study; SMC: Swedish Mammography Cohort; SMHS: Shanghai Men’s Health Study; SWHS: Shanghai Women’s Health Study; VITAL: VITamins And Lifestyle Study: Cohort Study of Dietary Supplements and Cancer Risk; WHI: Women’s Health Initiative; WLHS: Women’s Lifestyle and Health Study. ‡ Total participants included in unprocessed red meat analyses after applying exclusion criteria, with the exception of NLCS and CSDLH, which were analysed as case–cohort studies; for NLCS, a subcohort of 3653 males and 2479 females was randomly selected from 58,279 and 62,573 participants respectively; for CSDLH, a subcohort of 2560 males was randomly selected from 34,291. Females in CLUE II, CSDLH, JPHC I, JPHC II and SCHS were not included because the number of bladder cancer cases was lower than 50; EPIC included participants from 8 European countries: Denmark, France, Germany, Italy, the Netherlands, Spain, Sweden, and United Kingdom; for the Swedish Mammography Cohort, 1997 was considered the baseline because the questionnaire from that year included information on smoking habits, an important risk factor for bladder cancer, and only cases diagnosed from 1997 onward were included in the analysis. § Age at baseline and follow-up time are presented as median (10th–90th percentiles). Unprocessed red meat and processed meat intakes are presented as median (interquartile range), g/day. ‖ Number of participants excluded from the unprocessed red meat analysis. ^a^ SWHS was excluded from the processed meat analysis because of very low processed meat intake. The processed meat analysis included 2,502,700 participants (1,465,201 females and 1,037,499 males) and 20,672 bladder cancer cases (5199 females and 15,473 males). # Missing covariate data are presented as percentages of the baseline cohort size. Values greater than 0% but less than 0.1% are shown as <0.1.

## Data Availability

The sharing of data will be coordinated by the corresponding author and governed by executed data use agreements between the home institution of each cohort study and the Harvard T.H. Chan School of Public Health, as well as with approval from lead investigators or leadership group, for each cohort.

## Supplementary Materials

The following supporting information can be downloaded at: https://www.mdpi.com/article/10.3390/cancers18193203/s1, Figure S1: Flow chart for cohort and participant selection in the pooled analysis; Figure S2: Directed acyclic graph (DAG) showing assumed causal associations between processed meat and unprocessed red meat and bladder cancer risk; Figure S3: Median and interquartile range of intake of processed meat, by sex, for females (A) and males (B); Figure S4: Median and interquartile range of intake of unprocessed red meat, by sex, for females (A) and males (B); Figure S5: Minimally-adjusted associations between processed meat (A) and unprocessed red meat (B) intake and risk of bladder cancer, by sex; Figure S6: Study-specific multivariable-adjusted hazard ratios for bladder cancer comparing consumption of ≥40 g/day with <5 g/day of processed meat, by sex, for females (A) and males (B); Figure S7: Study-specific multivariable-adjusted hazard ratios for bladder cancer comparing consumption of ≥100 g/day with <20 g/day of unprocessed red meat, by sex, for females (A) and males (B); Figure S8: Associations between processed meat and unprocessed red meat consumption and risk of bladder cancer in all participants after excluding the first 2 years of follow-up; Figure S9: Associations between processed meat and unprocessed red meat consumption and risk of bladder cancer in all participants after restricting analyses to the 21 studies that systematically ascertained all types of bladder cancers; Figure S10: Associations between processed meat and unprocessed red meat consumption and risk of bladder cancer in all participants, after excluding data from sex-specific cohorts for which evidence of departure from proportional hazards was observed at *p* < 0.001; Table S1: Association between the consumption of processed meat and unprocessed red meat, and risk of bladder cancer, by geographic region; Table S2: Association between the consumption of processed meat and unprocessed red meat, and risk of bladder cancer, by race; Table S3: Association between consumption of processed meat and unprocessed red meat, and risk of bladder cancer, by follow-up time; Table S4: Association between consumption of processed meat and unprocessed red meat, and risk of bladder cancer, by attained age; Table S5: Minimally-adjusted and multivariable hazard ratios for bladder cancer risk associated with processed meat and unprocessed red meat consumption; Table S6: Multivariable hazard ratios for bladder cancer risk associated with processed meat and unprocessed red meat consumption among participants with complete data on all smoking variables; Table S7: Multivariable hazard ratios for bladder cancer risk associated with processed meat and unprocessed red meat consumption, restricted to participants in cohorts of teachers and health professionals and those with more than a high school education.

## Abbreviations

CI	confidence interval
DCPP	Pooling Project of Prospective Studies of Diet and Cancer
HR	hazard ratio
IARC	International Agency for Research on Cancer
ICD	International Classification of Diseases
NCI	National Cancer Institute
NOC	N-nitroso compounds
RR	relative risk
WCRF	World Cancer Research Fund
BCDDP	Breast Cancer Detection Demonstration Project Follow-Up Study
BWHS	Black Women’s Health Study
CARET	Beta-Carotene and Retinol Efficacy Trial
CLUE II	Campaign Against Cancer and Heart Disease
CNBSS	Canadian National Breast Screening Study
COSM	Cohort of Swedish Men
CPS II	Cancer Prevention Study II Nutrition Cohort
CSDLH	Canadian Study of Diet, Lifestyle and Health
CTS	California Teachers Study
EPIC	European Prospective Investigation into Cancer and Nutrition
HPFS	Health Professionals Follow-up Study (ClinicalTrials.gov Registration ID: NCT00005182; Registration URL: https://clinicaltrials.gov/study/NCT00005182 (accessed on 1 September 2026))
IWHS	Iowa Women’s Health Study
JPHC I	Japan Public Health Center-based Prospective Study I
JPHC II	Japan Public Health Center-based Prospective Study II
MCCS	Melbourne Collaborative Cohort Study
MEC	Multiethnic Cohort Study
NHS	Nurses’ Health Study
NHS II	Nurses’ Health Study II
NIH-AARP	NIH-AARP Diet and Health Study
NLCS	Netherlands Cohort Study
NYSC	New York State Cohort
PLCO	Prostate, Lung, Colorectal, and Ovarian Cancer Screening Trial
SCCS	Southern Community Cohort Study
SCHS	Singapore Chinese Health Study
SMC	Swedish Mammography Cohort
SMHS	Shanghai Men’s Health Study
SWHS	Shanghai Women’s Health Study
VITAL	VITamins And Lifestyle Study: Cohort Study of Dietary Supplements and Cancer Risk
WHI	Women’s Health Initiative
WLHS	Women’s Lifestyle and Health Study
