# Supplementary material for: Consumption of Processed Meat and Unprocessed Red Meat and Risk of Bladder Cancer: A Pooled Analysis of 30 Cohort Studies
Source: Cancers (Basel). 2026 Oct 3;18(19):3203. doi: 10.3390/cancers18193203 (PMC13664252; doi:10.3390/cancers18193203)
Supplement: Supplementary file 1 [file cancers-18-03203-s001.zip › Supplementary Figures.pdf]

Supplementary Figure S1. Flow Chart for cohort and participant selection in the pooled analysis.

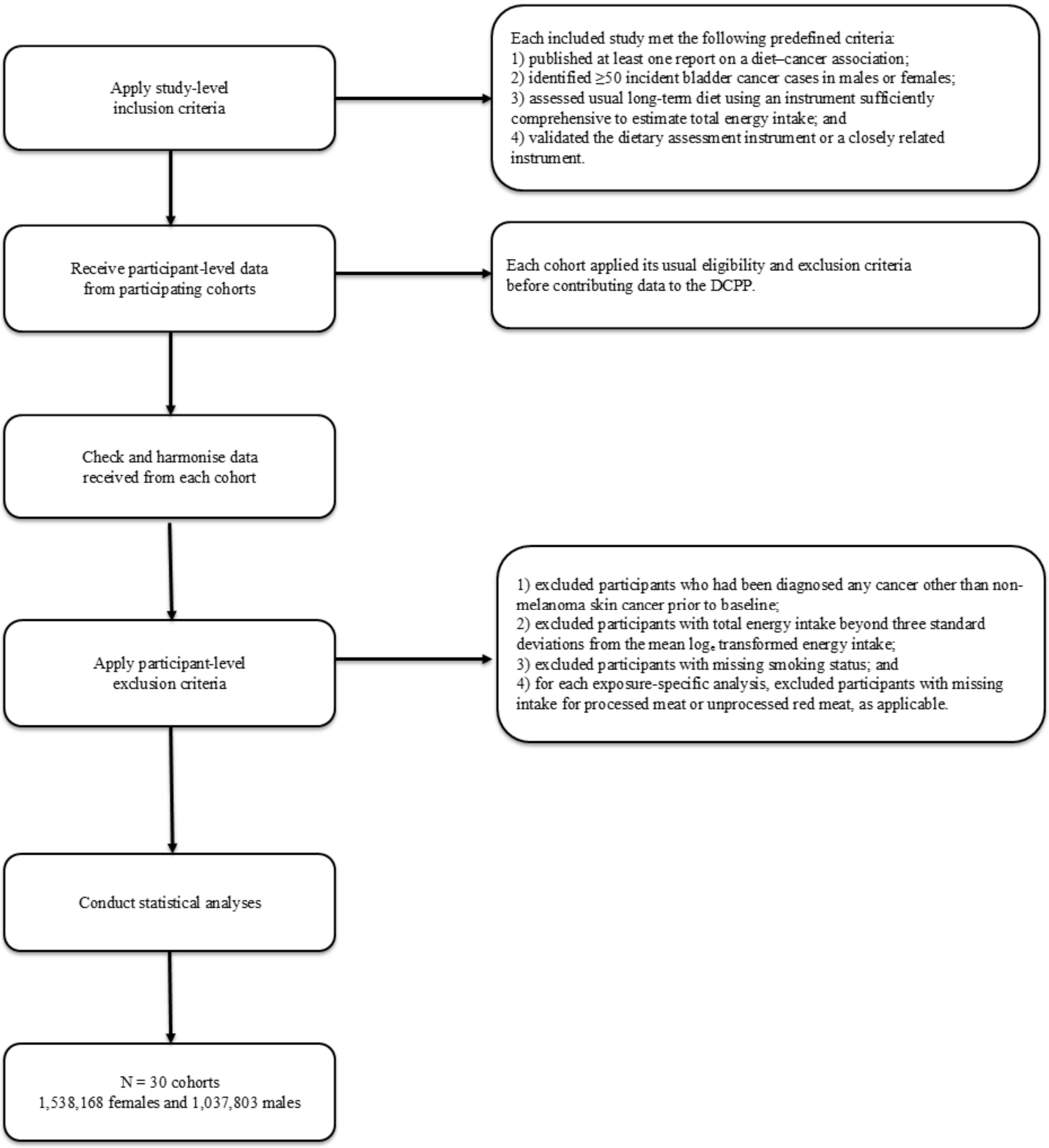

Supplementary Figure S2. Directed acyclic graph (DAG) showing assumed causal associations between processed meat and unprocessed red meat and bladder cancer risk.

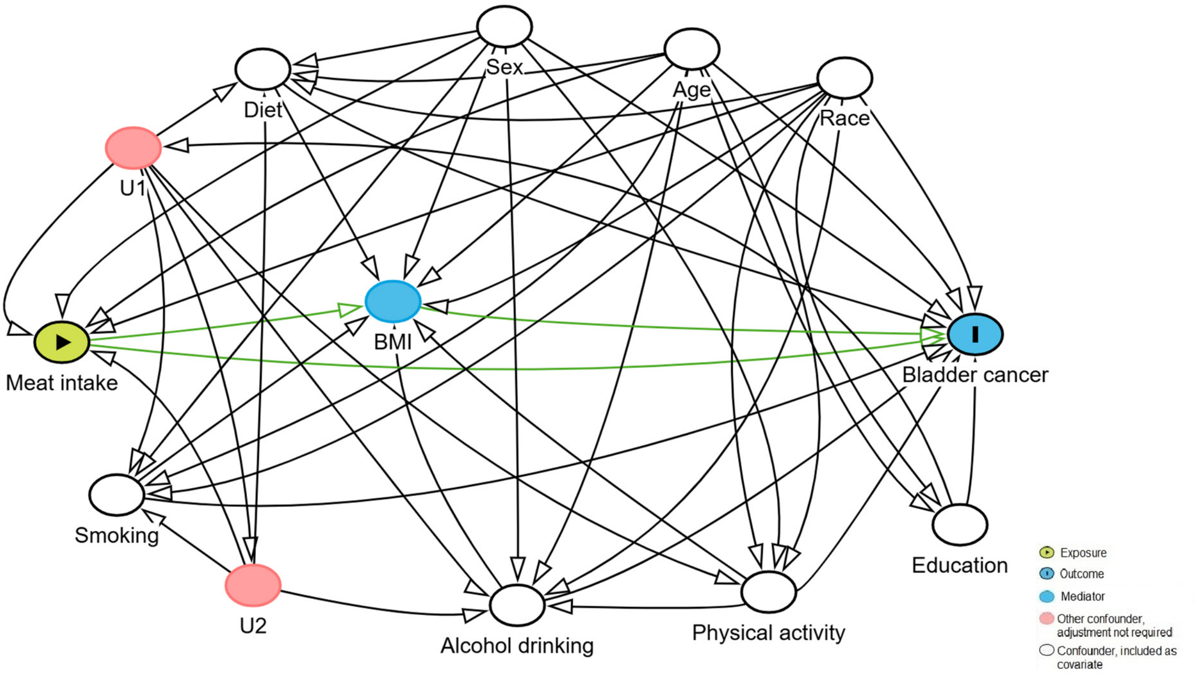

U1: Health consciousness, U2: Behavioural factor; Green arcs represent the causal pathway(s) of interest; BMI was not adjusted for so that the total effect of meat intake on bladder cancer risk could be estimated.

Diet includes total fruit intake (including fruit juice, g/day), total vegetable intake (including vegetable juice, g/day), and total energy intake (kcal/day), all modelled as continuous variables. In the main models, processed and unprocessed red meat were mutually adjusted for each other.

Supplementary Figure S3. Median and interquartile range of intake of processed meat, by sex, for females (A) and males (B).

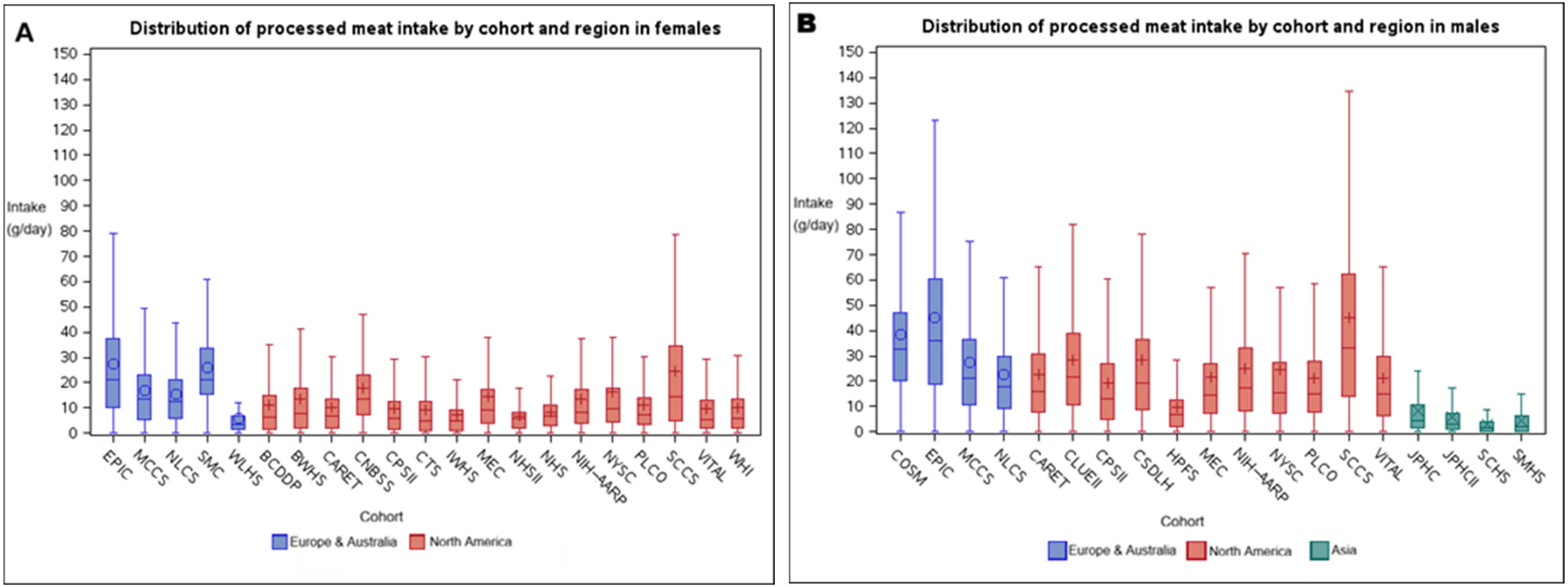

Supplementary Figure S4. Median and interquartile range of intake of unprocessed red meat, by sex, for females (A) and males (B).

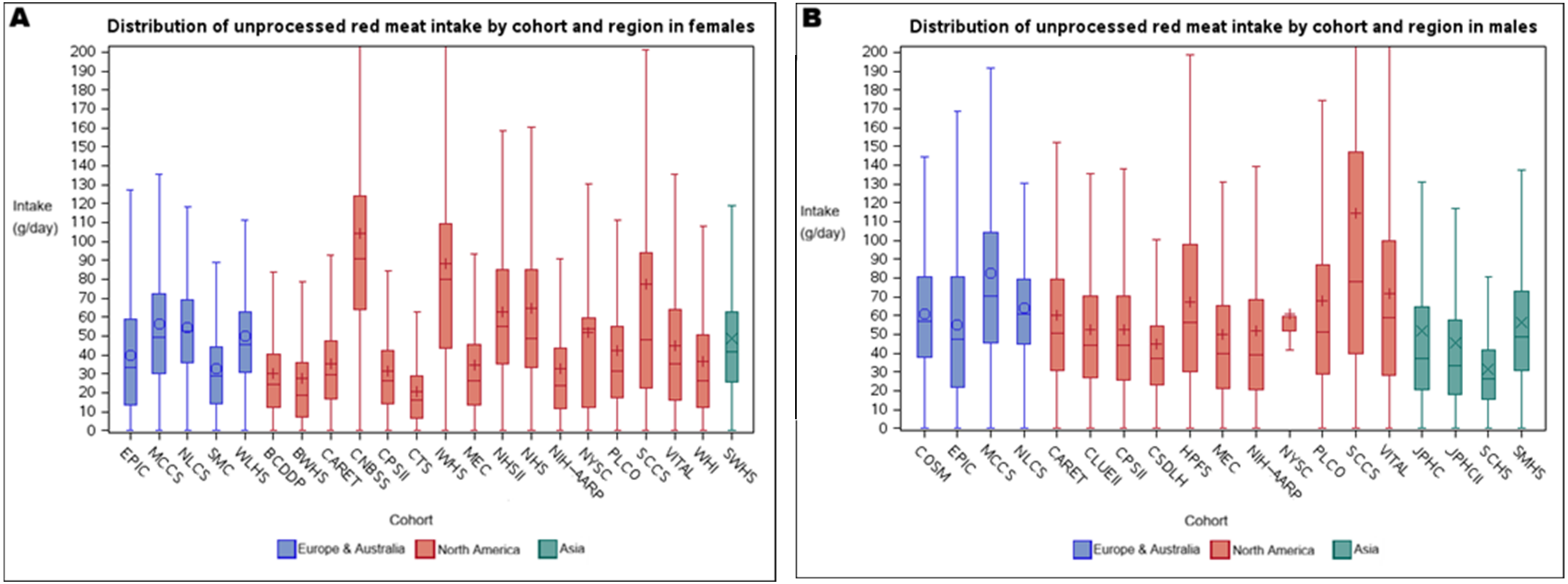

Supplementary Figure S5. Minimally-adjusted associations between processed meat (A) and unprocessed red meat (B) intake and risk of bladder cancer, by sex.

A

	Cases	HR (95% CI)*	P-study[a]	I²	P-trend[b]	P-sex[c]
**Females**						
< 5 g/day	1,737	1.00				
5-9.9 g/day	1,140	1.05 (0.97-1.13)				
10-19.9 g/day	1,167	1.07 (0.99-1.15)				
20-39.9 g/day	817	1.09 (0.99-1.20)				
≥ 40 g/day	338	1.17 (1.03-1.34)	0.90	0%	0.03	
Continuous (per 20 g/day)	5,199	1.04 (1.00-1.07)	0.66	0%		
**Males**						
< 5 g/day	2,666	1.00				
5-9.9 g/day	2,319	1.08 (1.02-1.14)				
10-19.9 g/day	3,655	1.11 (1.05-1.17)				
20-39.9 g/day	3,960	1.14 (1.06-1.22)				
≥ 40 g/day	2,873	1.19 (1.12-1.27)	0.97	0%	<0.001	
Continuous (per 20 g/day)	15,473	1.03 (1.02-1.05)	0.87	0%		
**Combined**						
< 5 g/day	4,403	1.00				
5-9.9 g/day	3,459	1.07 (1.02-1.12)				
10-19.9 g/day	4,822	1.09 (1.05-1.14)				
20-39.9 g/day	4,777	1.13 (1.07-1.19)				
≥ 40 g/day	3,211	1.19 (1.12-1.26)	0.99	0%	<0.001	0.84
Continuous (per 20 g/day)	20,672	1.03 (1.02-1.05)	0.89	0%		0.94

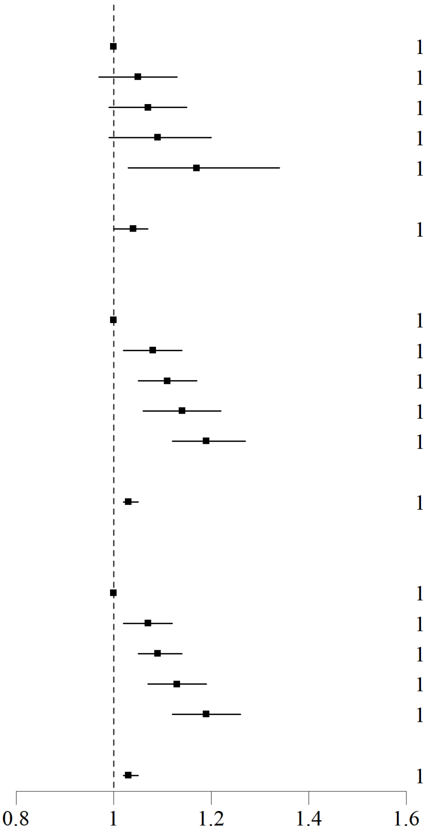

B

	Cases	HR(95% CI)*	P-study[a]	I²	P-trend[b]	P-sex[c]
**Females**						
< 20 g/day	1,672	1.00				
20-39.9 g/day	1,454	1.04 (0.96-1.11)				
40-59.9 g/day	912	1.04 (0.95-1.14)				
60-99.9 g/day	811	1.04 (0.94-1.15)				
≥ 100 g/day	448	1.09 (0.92-1.28)	0.24	17%	0.49	
Continuous (per 50 g/day)	5,297	1.02 (0.98-1.07)	0.15	25%		
**Males**						
< 20 g/day	2,822	1.00				
20-39.9 g/day	3,691	1.05 (0.96-1.14)				
40-59.9 g/day	3,287	1.11 (1.05-1.17)				
60-99.9 g/day	3,677	1.14 (1.08-1.20)				
≥ 100 g/day	2,006	1.16 (1.09-1.24)	0.70	0%	<0.001	
Continuous (per 50 g/day)	15,483	1.03 (1.01-1.05)	0.36	9%		
**Combined**						
< 20 g/day	4,494	1.00				
20-39.9 g/day	5,145	1.04 (0.98-1.10)				
40-59.9 g/day	4,199	1.09 (1.04-1.14)				
60-99.9 g/day	4,488	1.12 (1.06-1.17)				
≥ 100 g/day	2,454	1.15 (1.08-1.22)	0.45	1%	0.001	0.33
Continuous (per 50 g/day)	20,780	1.03 (1.01-1.05)	0.20	17%		0.61

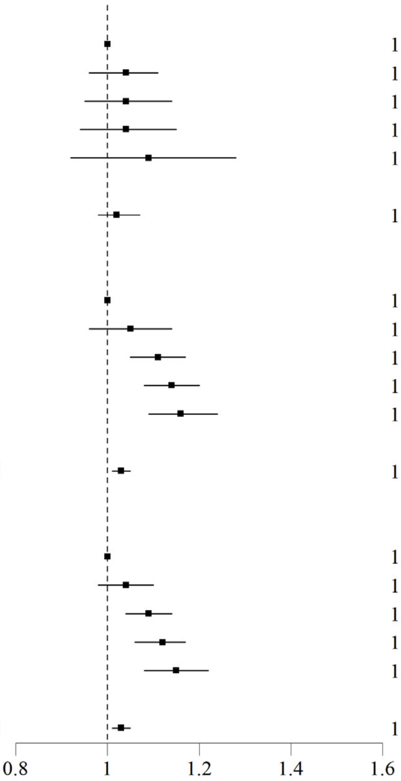

HR, hazard ratio; CI, confidence interval

* All models were stratified jointly on baseline age (in years), year of baseline questionnaire completion, and centre (EPIC only).

[a] Between-study heterogeneity was tested using the Q-statistic.

[b] Linear trend was tested using a continuous variable based on the median of each category.

[c] Heterogeneity by sex was examined using meta-regression.

Supplementary Figure S6. Study-specific multivariable-adjusted hazard ratios for bladder cancer comparing consumption of ⩾ 40 g/day with <5 g/day of processed meat, by sex, for females (A) and males (B).

A

	Cases	HR (95% CI)*	P-study[a]	I²
**Females**				
BCDDP	2	0.48 (0.11-2.09)		
BWHS	4	1.16 (0.36-3.80)		
CARET	1	0.29 (0.02-3.37)		
CNBSS	15	0.97 (0.51-1.82)		
CPS II	10	0.85 (0.44-1.65)		
CTS	11	0.88 (0.46-1.67)		
EPIC	110	1.00 (0.71-1.42)		
IWHS	4	1.58 (0.56-4.44)		
MCCS	9	1.16 (0.53-2.54)		
MEC	31	1.22 (0.78-1.89)		
NHS	3	1.29 (0.40-4.11)		
NIH-AARP	61	1.12 (0.83-1.51)		
NLCS	14	1.09 (0.57-2.08)		
NYSC	4	0.44 (0.13-1.42)		
PLCO	7	2.18 (0.91-5.23)		
SCCS	12	2.26 (0.88-5.78)		
SMC	14	1.19 (0.43-3.28)		
VITAL	5	1.63 (0.61-4.34)		
WHI	21	1.34 (0.83-2.17)		
**Pooled HR**	**338**	**1.12 (0.97-1.29)**	**0.80**	**0%**

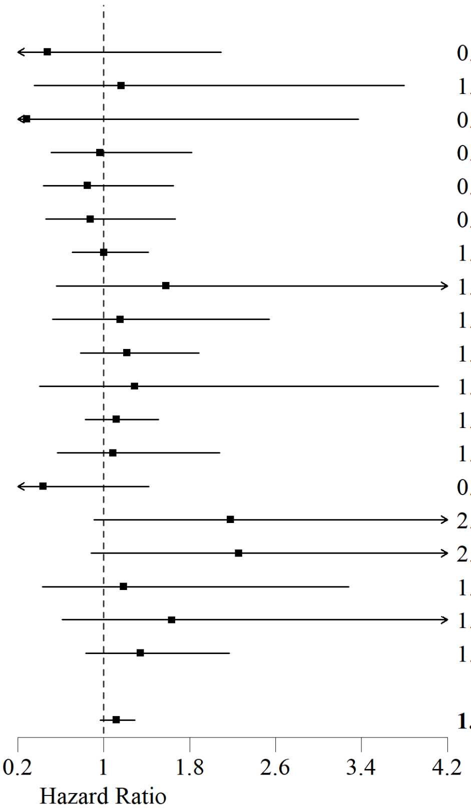

B

	Cases	HR (95% CI)*	P-study[a]	I²
**Males**				
CARET	53	0.89 (0.58-1.36)		
CLUE II	26	1.35 (0.67-2.71)		
COSM	268	0.92 (0.58-1.44)		
CPS II	196	1.05 (0.86-1.27)		
CSDLH	27	1.08 (0.52-2.23)		
EPIC	604	1.20 (0.91-1.58)		
HPFS	17	1.26 (0.76-2.09)		
JPHC I	1	0.53 (0.06-4.55)		
JPHC II	1	1.21 (0.16-8.98)		
MCCS	84	1.04 (0.73-1.46)		
MEC	186	1.17 (0.94-1.45)		
NIH-AARP	1,000	1.06 (0.95-1.18)		
NLCS	201	1.14 (0.84-1.53)		
NYSC	37	1.07 (0.63-1.82)		
PLCO	75	1.10 (0.77-1.57)		
SCCS	43	0.71 (0.39-1.27)		
SMHS	2	1.49 (0.36-6.21)		
VITAL	52	1.01 (0.68-1.50)		
**Pooled HR**	**2,873**	**1.07 (1.00-1.15)**	**0.99**	**0%**

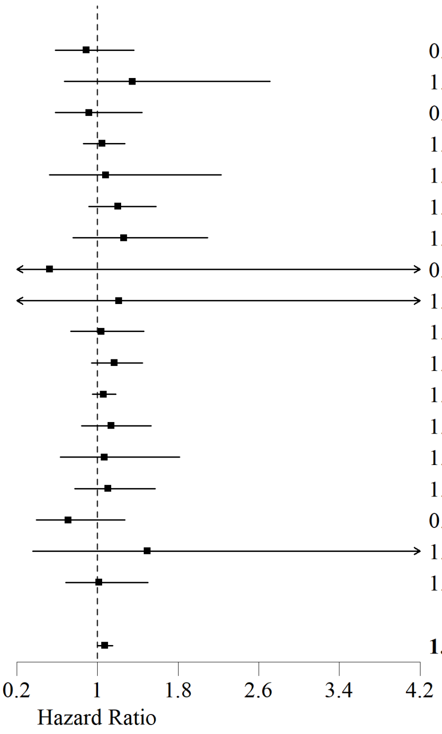

HR, hazard ratio; CI, confidence interval

* All models were stratified jointly on baseline age (in years), year of baseline questionnaire completion, and centre (EPIC only)and adjusted for race and ethnicity, smoking status and duration, educational level, alcohol consumption, physical activity, unprocessed red meat intake, total fruit (including fruit juice) intake, total vegetable (including vegetable juice) intake, and total energy intake; these variables were entered directly into the multivariable model or, for studies with less than 200 cases, modelled using propensity scores.

[a] Between-study heterogeneity was tested using the Q-statistic.

Supplementary Figure S7. Study-specific multivariable-adjusted hazard ratios for bladder cancer comparing consumption of ≥ 100 g/day with <20 g/day of unprocessed red meat, by sex, for females (A) and males (B).

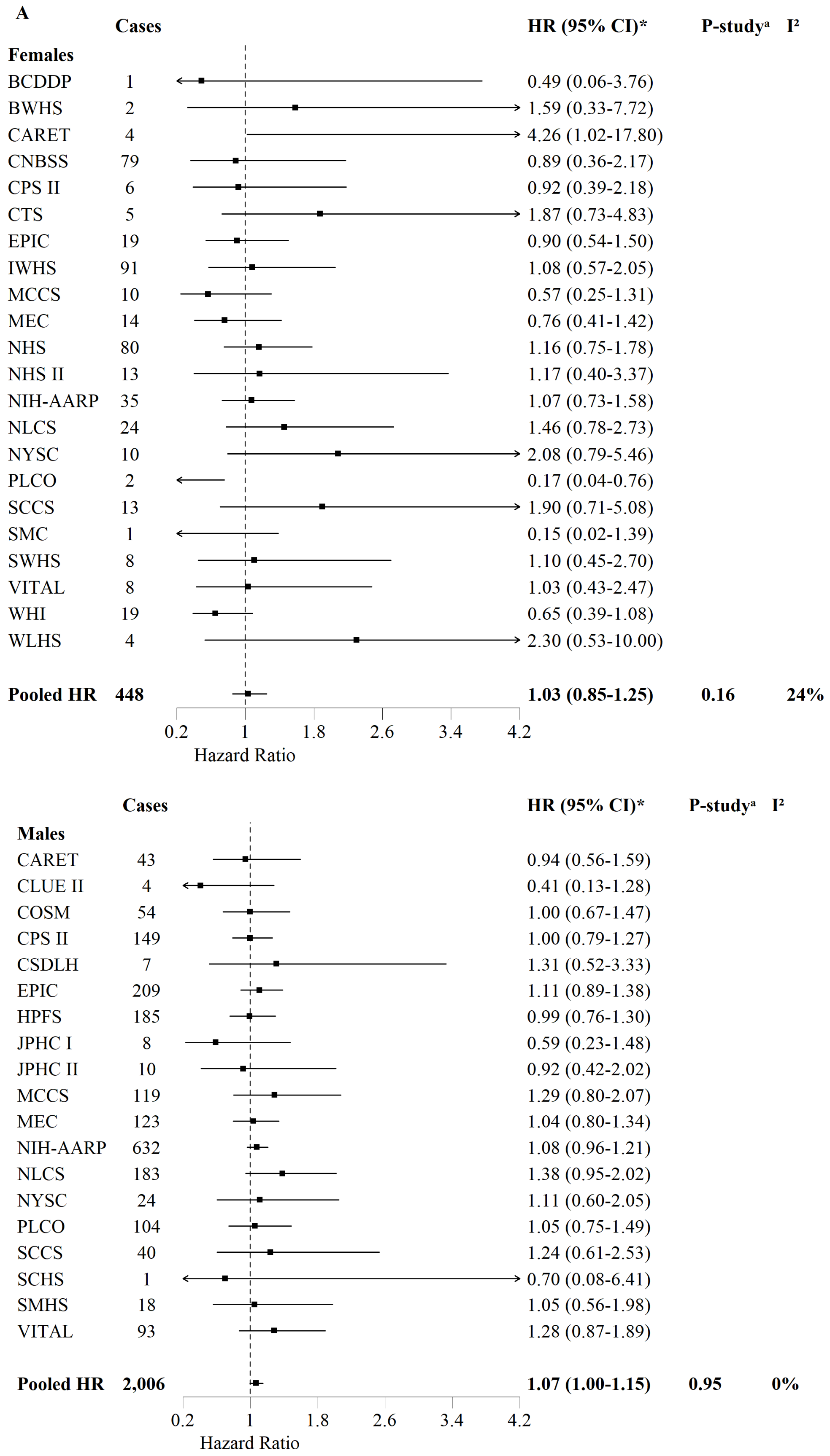

HR, hazard ratio; CI, confidence interval

* All models were stratified jointly on baseline age (in years), year of baseline questionnaire completion, and centre (EPIC only)and adjusted for race and ethnicity, smoking status and duration, educational level, alcohol consumption, physical activity, processed meat intake, total fruit (including fruit juice) intake, total vegetable (including vegetable juice) intake, and total energy intake; these variables were entered directly into the multivariable model or, for studies with less than 200 cases, modelled using propensity scores.

[a] Between-study heterogeneity was tested using the Q-statistic.

Supplementary Figure S8. Associations between processed meat and unprocessed red meat consumption and risk of bladder cancer in all participants after excluding the first 2 years of follow-up.

	Cases	HR (95% CI)*	P-study[a]	I^2	P-trend[b]
**Processed meat**					
< 5 g/day	4,022	1.00			
5-9.9 g/day	3,175	1.04 (0.99-1.09)			
10-19.9 g/day	4,457	1.05 (1.00-1.10)			
20-39.9 g/day	4,350	1.06 (1.00-1.12)			
≥ 40 g/day	2,917	1.10 (1.03-1.17)	0.98	0%	0.007
Continuous (per 20 g/day)	18,921	1.02 (1.01-1.04)	0.84	0%	
**Unprocessed red meat**					
< 20 g/day	4,083	1.00			
20-39.9 g/day	4,719	1.01 (0.96-1.06)			
40-59.9 g/day	3,848	1.03 (0.98-1.09)			
60-99.9 g/day	4,123	1.04 (0.98-1.09)			
≥ 100 g/day	2,248	1.07 (1.00-1.15)	0.64	0%	0.13
Continuous (per 50 g/day)	19,021	1.01 (0.99-1.03)	0.33	8%	

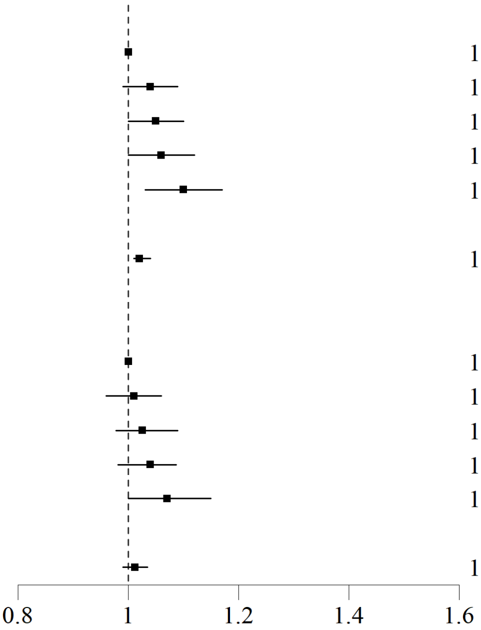

HR, hazard ratio; CI, confidence interval

* All models were stratified jointly on baseline age (in years), year of baseline questionnaire completion, and centre (EPIC only)and adjusted for race and ethnicity, smoking status and duration, educational level, alcohol consumption, physical activity, analysis for processed meat were additionally adjusted for unprocessed red meat and vice visa, total fruit (including fruit juice) intake, total vegetable (including vegetable juice) intake, and total energy intake; these variables were entered directly into the multivariable model or, for studies with less than 200 cases, modelled using propensity scores.

[a] Between-study heterogeneity was tested using the Q-statistic.

[b] Linear trend was tested using a continuous variable based on the median of each category.

Supplementary Figure S9. Associations between processed meat and unprocessed red meat consumption and risk of bladder cancer in all participants after restricting analyses to the 21 studies that systematically ascertained all types of bladder cancers.

	Cases	HR (95% CI)*	P-study[a]	I^2	P-trend[b]
**Processed meat**					
< 5 g/day	3,866	1.00			
5-9.9 g/day	3,263	1.02 (0.97-1.07)			
10-19.9 g/day	4,603	1.03 (0.98-1.08)			
20-39.9 g/day	4,648	1.06 (1.01-1.12)			
≥ 40 g/day	3,124	1.09 (1.02-1.16)	0.97	0%	0.009
Continuous (per 20 g/day)	19,504	1.02 (1.00-1.03)	0.89	0%	
**Unprocessed red meat**					
< 20 g/day	4,228	1.00			
20-39.9 g/day	4,890	1.00 (0.95-1.06)			
40-59.9 g/day	3,857	1.02 (0.97-1.08)			
60-99.9 g/day	4,254	1.04 (0.98-1.09)			
≥ 100 g/day	2,285	1.06 (0.98-1.14)	0.38	6%	0.32
Continuous (per 50 g/day)	19,514	1.01 (0.98-1.03)	0.32	10%	

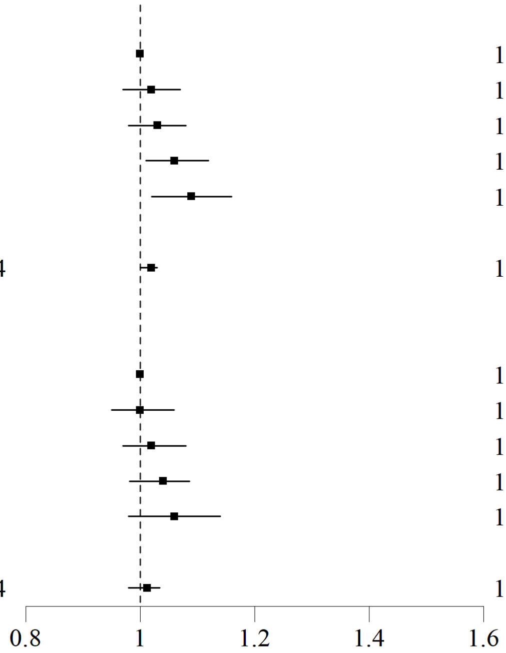

HR, hazard ratio; CI, confidence interval

* All models were stratified jointly on baseline age (in years), year of baseline questionnaire completion, and centre (EPIC only)and adjusted for race and ethnicity, smoking status and duration, educational level, alcohol consumption, physical activity, analysis for processed meat were additionally adjusted for unprocessed red meat and vice visa, total fruit (including fruit juice) intake, total vegetable (including vegetable juice) intake, and total energy intake; these variables were entered directly into the multivariable model or, for studies with less than 200 cases, modelled using propensity scores.

[a] Between-study heterogeneity was tested using the Q-statistic.

[b] Linear trend was tested using a continuous variable based on the median of each category.

Supplementary Figure S10. Associations between processed meat and unprocessed red meat consumption and risk of bladder cancer in all participants, after excluding data from sex-specific cohorts for which evidence of departure from proportional hazards was observed at P<0.001.

	Cases	HR (95% CI)*	P-study[a]	I²	P-trend[b]
**Processed meat**					
< 5 g/day	4,264	1.00			
5-9.9 g/day	3,330	1.02 (0.97-1.07)			
10-19.9 g/day	4,700	1.03 (0.98-1.08)			
20-39.9 g/day	4,708	1.05 (1.00-1.11)			
≥ 40 g/day	3,180	1.08 (1.01-1.15)	0.99	0%	0.009
Continuous (per 20 g/day)	20,182	1.02 (1.00-1.03)	0.90	0%	
**Unprocessed red meat**					
< 20 g/day	4,057	1.00			
20-39.9 g/day	4,542	0.98 (0.92-1.04)			
40-59.9 g/day	3,782	1.02 (0.97-1.07)			
60-99.9 g/day	4,129	1.04 (0.98-1.09)			
≥ 100 g/day	2,291	1.07 (1.00-1.15)	0.59	0%	0.06
Continuous (per 50 g/day)	18,801	1.02 (0.99-1.04)	0.31	10%	

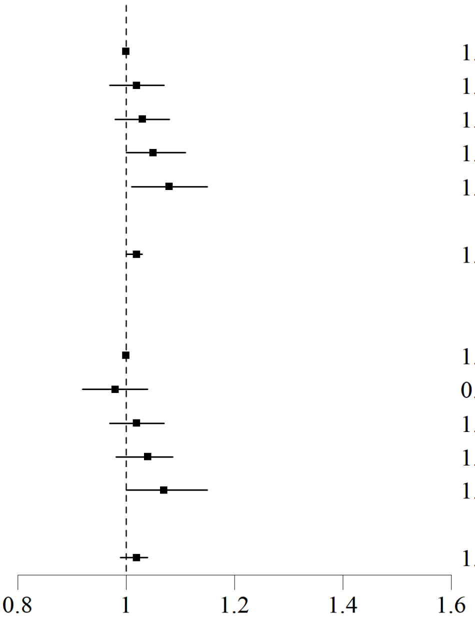

HR, hazard ratio; CI, confidence interval

* All models were stratified jointly on baseline age (in years), year of baseline questionnaire completion, and centre (EPIC only)and adjusted for race and ethnicity, smoking status and duration, educational level, alcohol consumption, physical activity, analysis for processed meat were additionally adjusted for unprocessed red meat and vice visa, total fruit (including fruit juice) intake, total vegetable (including vegetable juice) intake, and total energy intake; these variables were entered directly into the multivariable model or, for studies with less than 200 cases, modelled using propensity scores.

[a] Between-study heterogeneity was tested using the Q-statistic.

[b] Linear trend was tested using a continuous variable based on the median of each category.
