# Supplementary material for: Consumption of Processed Meat and Unprocessed Red Meat and Risk of Bladder Cancer: A Pooled Analysis of 30 Cohort Studies
Source: Cancers (Basel). 2026 Oct 3;18(19):3203. doi: 10.3390/cancers18193203 (PMC13664252; doi:10.3390/cancers18193203)
Supplement: Supplementary file 1 [file cancers-18-03203-s001.zip › Supplementary Tables.pdf]

Supplementary Table S1. Association between the consumption of processed meat and unprocessed red meat, and risk of bladder cancer, by geographic region.

	NORTH AMERICA[#]		***ASIA***[#]		***EUROPE & AUSTRALIA***[#]		
	No. of cases	***HR (95% CI)*** [*]	***No. of cases***	***HR (95% CI)*** [*]	***No. of cases***	***HR (95% CI)*** [*]	***P-het***[※]
Processed meat							
Per 20 g/day increment	14,984	1.02 (1.00-1.04)	559	1.08 (0.89-1.31)	5,129	1.01 (0.99-1.04)	0.90
⩾40 g/day vs. <5 g/day intake	1,903	1.08 (1.01-1.16)	4	¶	1,304	1.09 (0.95-1.25)	0.53
Unprocessed red meat							
Per 50 g/day increment	14,983	1.00 (0.98-1.03)	657	1.00 (0.89-1.13)	5,140	1.03 (0.97-1.10)	0.76
≥100 g/day vs. <20 g/day	1,786	1.06 (0.98-1.14)	45	¶	623	1.11 (0.93-1.33)	0.62

HR, hazard ratio; CI, confidence interval

# Geographic region was coded as North America (BCDDP, BWHS, CARET, CLUE II, CNBSS, CPS II, CSDLH, CTS, HPFS, IWHS, MEC, NHS, NHS II, NIH-AARP, NYSC, PLCO, SCCS, VITAL, WHI), Europe and Australia (COSM, EPIC, MCCS, NLCS, SMC, WLHS), and Asia (JPHC I, JPHC II, SCHS, SMHS, SWHS).

* All models were stratified jointly on baseline age (in years), year of baseline questionnaire completion, and centre (EPIC only) and adjusted for race and ethnicity, smoking status and duration, educational level, alcohol consumption, physical activity, analysis for processed meat were additionally adjusted for unprocessed red meat and vice visa, total fruit (including fruit juice) intake, total vegetable (including vegetable juice) intake, and total energy intake; these variables were entered directly into the multivariable model or, for studies with less than 200 cases, modelled using propensity scores.

¶ HR estimates for the highest versus lowest consumption categories of processed and unprocessed red meat in Asians are not reported due to there being fewer than 50 cases in the highest category.

※ Heterogeneity by geographic region was evaluated using meta-regression.

Supplementary Table S2. Association between the consumption of processed meat and unprocessed red meat, and risk of bladder cancer, by race.

	Asian#		*Black#*		*White*		
	No. of cases	***HR (95% CI) ****	***No. of cases***	***HR (95% CI) ****	***No. of cases***	***HR (95% CI) ****	***P-het※***
Processed meat							
Per 20 g/day increment	1280	1.07 (0.99-1.16)	605	1.02 (0.96-1.09)	18,001	1.01 (1.00-1.03)	0.37
≥40 g/day vs. <5 g/day intake	61	1.22 (0.90-1.65)	102	1.51 (1.10-2.08)	2,953	1.04 (0.98-1.11)	
Unprocessed red meat							
Per 50 g/day increment	1378	0.94 (0.85-1.03)	605	1.03 (0.93-1.13)	18,011	1.01 (0.99-1.03)	0.15
≥100 g/day vs. <20 g/day	78	0.92 (0.69-1.23)	48	¶	2,216	1.06 (0.99-1.13)	

HR, hazard ratio; CI, confidence interval

# Studies contributing bladder cancer cases to the aggregated analyses of processed meat among individuals of Asian race: BCDDP, CARET, CPS II, CSDLH, CTS, HPFS, IWHS, JPHC I, JPHC II, MEC, NHS, NIH-AARP, PLCO, SCHS, SMHS, VITAL, and WHI. Studies contributing to the aggregated analyses of unprocessed red meat among individuals of Asian race: BCDDP, CARET, CPS-II, CSDLH, CTS, HPFS, IWHS, JPHC I, JPHC II, MEC, NHS, NIH-AARP, PLCO, SCHS, SMHS, SWHS, VITAL, and WHI. Studies contributing to the aggregated analyses of processed meat and unprocessed red meat among individuals of Black race: BCDDP, BWHS, CARET, CPS II, CTS, HPFS, IWHS, MEC, NHS, NHS, NIH-AARP, NYSC, PLCO, SCCS, VITAL and WHI.

* All models were stratified jointly on baseline age (in years), year of baseline questionnaire completion, and centre (EPIC only) and adjusted for smoking status and duration, educational level, alcohol consumption, physical activity, analysis for processed meat were additionally adjusted for unprocessed red meat and vice visa, total fruit (including fruit juice) intake, total vegetable (including vegetable juice) intake, and total energy intake; these variables were entered directly into the multivariable model.

¶ HR estimates for the highest versus lowest consumption categories of unprocessed red meat in Black participants are not reported due to there being fewer than 50 cases in the highest category.

※ P-values for heterogeneity by race were obtained by comparing the log-likelihoods of continuous models with and without interaction terms, based on a χ^2 distribution with two degrees of freedom.

Supplementary Table S3. Association between consumption of processed meat and unprocessed red meat, and risk of bladder cancer, by follow-up time.

	<5 years		***5-9.9 years***		***≥10 years***		
	No. of cases	***HR (95% CI) ****	***No. of cases***	***HR (95% CI) ****	***No. of cases***	***HR (95% CI) ****	***p-het***[※]
Processed meat							
Per 20 g/day increment	4,807[¶]	1.01 (0.98-1.04)	5,674[†]	1.07 (1.03-1.11)	9,916[‡‡]	1.01 (0.99-1.03)	0.02
≥40 g/day vs. <5 g/day intake	794[¶]	1.08 (0.95-1.22)	1,012[†]	1.24 (1.09-1.41)	1,376[‡‡]	1.01 (0.92-1.11)	0.03
Unprocessed red meat							
Per 50 g/day increment	4,812[††]	1.03 (0.98-1.07)	5,706[†]	1.02 (0.97-1.07)	9,968[‡‡]	1.01 (0.98-1.04)	0.70
≥100 g/day vs. <20 g/day	556[††]	1.09 (0.95-1.24)	686[†]	1.13 (1.00-1.28)	1,178[‡‡]	1.02 (0.93-1.13)	0.48

HR, hazard ratio; CI, confidence interval

* All models were stratified jointly on baseline age (in years), year of baseline questionnaire completion, and centre (EPIC only) and adjusted for race and ethnicity, smoking status and duration, educational level, alcohol consumption, physical activity, analysis for processed meat were additionally adjusted for unprocessed red meat and vice visa, total fruit (including fruit juice) intake, total vegetable (including vegetable juice) intake, and total energy intake; these variables were entered directly into the multivariable model or, for studies with less than 200 cases, modelled using propensity scores.

※ Heterogeneity by follow-up time was evaluated using meta-regression.

¶ BWHS, CARET (female), CLUE II, CNBSS, JPHC I, MEC (female), NHS II, SCCS (female) and WLHS were excluded from the analysis for processed meat due to them having fewer than 25 cases in this stratum.

†† BWHS, CARET (female), CLUE II, CNBSS, JPHC I, MEC (female), NHS II, SCCS (female), SWHS and WLHS were excluded from the analysis for unprocessed red meat due to them having fewer than 25 cases in this stratum

† BWHS, CARET (female), CLUE II, JPHC I, NHS II, NYSC (female) and WLHS were excluded from the analysis due to them having fewer than 25 cases in this stratum.

‡‡ CSDLH, PLCO, SCCS (female) and VITAL were excluded from the analysis due to them having fewer than 25 cases in this stratum.

Supplementary Table S4. Association between consumption of processed meat and unprocessed red meat, and risk of bladder cancer, by attained age[†].

	<70 years		*70-74.9 years*		*≥75 years*		
	No. of cases	***HR (95% CI) ****	***No. of cases***	***HR (95% CI) ****	***No. of cases***	***HR (95% CI) ****	***P-het*[※]**
Processed meat							
Per 20 g/day increment	7,915[¶]	1.02 (1.00-1.04)	4,860[†]	1.02 (0.99-1.05)	7,581[‡‡]	1.03 (1.00-1.06)	0.72
≥40 g/day vs. <5 g/day intake	1,476[¶]	1.07 (0.97-1.19)	761[†]	1.14 (1.00-1.30)	941[‡‡]	1.08 (0.97-1.19)	0.69
Unprocessed red meat							
Per 50 g/day increment	7,971[¶]	1.04 (1.02-1.07)	4,860[††]	1.01 (0.96-1.07)	7,590[§]	0.98 (0.94-1.03)	0.02
≥100 g/day vs. <20 g/day intake	1,162[¶]	1.15 (1.04-1.27)	563[††]	1.14 (0.95-1.35)	698[§]	0.97 (0.85-1.11)	0.08

HR, hazard ratio; CI, confidence interval

[†] Attained age refers to the participant's age during follow-up, calculated as age at baseline plus time since study entry.

* All models were stratified jointly on baseline age (in years), year of baseline questionnaire completion, and centre (EPIC only) and adjusted race and ethnicity, smoking status and duration, educational level, alcohol consumption, physical activity, analysis for processed meat were additionally adjusted for unprocessed red meat and vice visa, total fruit (including fruit juice) intake, total vegetable (including vegetable juice) intake, and total energy intake; these variables were entered directly into the multivariable model or, for studies with less than 200 cases, modelled using propensity scores.

[※] Heterogeneity by age was evaluated using meta-regression.

¶ CSDLH and NYSC (females) were excluded from the analysis due to them having fewer than 25 cases in this stratum.

† BCDDP, BWHS, CARET (females), CLUE II, CSDLH, NYSC (females), NHS II, SCCS, SCHS, SMC, VITAL (females) and WLHS were excluded from the analysis for processed meat due to them having fewer than 25 cases in this stratum.

†† BCDDP, BWHS, CARET (females), CLUE II, CSDLH, NYSC (females), NHS II, SCCS, SCHS, SWHS, SMC, VITAL (females) and WLHS were excluded from the analysis for unprocessed red meat due to them having fewer than 25 cases in this stratum.

‡‡ BWHS, CARET (females), CNBSS, JPHC I, NHS II, NYSC (females) and SCHS were excluded from the analysis for processed meat due to them having fewer than 25 cases in this stratum.

§ BWHS, CARET (females), CNBSS, JPHC I, NHS II, NYSC (females), SWHS and SCHS were excluded from the analysis for processed meat due to them having fewer than 25 cases in this stratum.

Supplementary Table S5. Minimally-adjusted and multivariable hazard ratios for bladder cancer risk associated with processed meat and unprocessed red meat consumption.

	MEAT INTAKE (grams/day)					Continuous, per 20 g/d increment
	<5 (Ref.)	5-9.9	10-19.9	20-39.9	≥40	
	n=3,282	n=2,305	n=3,204	n=3,128	n=2,122	n=14,041
**Processed meat (25 cohorts)**	HR (95% CI)	HR (95% CI)	HR (95% CI)	HR (95% CI)	HR (95% CI)	HR (95% CI)
Model A: minimally-adjusted*	1.00	1.04 (0.98-1.10)	1.08 (1.02-1.14)	1.10 (1.03-1.17)	1.17 (1.09-1.26)	1.03 (1.02-1.05)
Model A + smoking status	1.00	1.01 (0.95-1.06)	1.03 (0.98-1.09)	1.03 (0.97-1.09)	1.07 (0.99-1.15)	1.01 (1.00-1.03)
Model A + smoking status and duration	1.00	1.00 (0.94-1.06)	1.02 (0.97-1.08)	1.02 (0.96-1.08)	1.05 (0.98-1.13)	1.01 (0.99-1.03)
Model A + all covariates** (excluding smoking)	1.00	1.02 (0.97-1.08)	1.06 (1.00-1.12)	1.08 (1.01-1.15)	1.17 (1.08-1.26)	1.04 (1.02-1.05)
Model A + all covariates** + smoking status	1.00	1.01 (0.95-1.06)	1.03 (0.98-1.09)	1.04 (0.97-1.11)	1.10 (1.02-1.19)	1.02 (1.00-1.04)
Model A + all covariates** + smoking status and duration ‡	1.00	1.00 (0.95-1.06)	1.03 (0.97-1.09)	1.03 (0.97-1.10)	1.09 (1.00-1.18)	1.02 (1.00-1.04)

	<20 (Ref.)	20-39.9	40-59.9	60-99.9	≥100	Continuous, per 50 g/day increment
	n=2,732	n=3,336	n=2,980	n=3,336	n=1,765	n=14,149
**Unprocessed red meat (26 cohorts)**	HR (95% CI)	HR (95% CI)	HR (95% CI)	HR (95% CI)	HR (95% CI)	HR (95% CI)
Model A: minimally-adjusted*	1.00	1.04 (0.97-1.11)	1.10 (1.03-1.17)	1.10 (1.03-1.17)	1.12 (1.04-1.21)	1.03 (1.00-1.05)
Model A + smoking status	1.00	1.01 (0.95-1.08)	1.04 (0.98-1.10)	1.04 (0.98-1.10)	1.04 (0.96-1.12)	1.01 (0.98-1.03)
Model A + smoking status and duration	1.00	1.01 (0.95-1.07)	1.03 (0.97-1.09)	1.03 (0.97-1.09)	1.03 (0.95-1.10)	1.01 (0.98-1.03)
Model A + all covariates** (excluding smoking)	1.00	1.01 (0.95-1.08)	1.06 (0.99-1.12)	1.07 (1.00-1.14)	1.11 (1.03-1.21)	1.03 (1.00-1.06)
Model A + all covariates** + smoking status	1.00	1.00 (0.94-1.07)	1.04 (0.98-1.10)	1.04 (0.97-1.11)	1.07 (0.99-1.16)	1.02 (0.99-1.04)
Model A + all covariates** + smoking status and duration ‡	1.00	1.00 (0.94-1.07)	1.03 (0.97-1.10)	1.03 (0.97-1.10)	1.07 (0.98-1.16)	1.02 (0.99-1.04)

HR, hazard ratio; CI, confidence interval; n, number of bladder cancer cases

*Model A was stratified jointly on baseline age (in years), year of baseline questionnaire completion, and centre (EPIC only).

**Covariates recorded at baseline were included in multivariable models: race and ethnicity, alcohol drinking, physical activity, processed meat intake (only in unprocessed red meat analyses), total fruit (including fruit juice) intake, total vegetable (including vegetable juice) intake and total energy intake. These variables were entered directly into the multivariable model or, for studies with less than 200 cases, these variables were modelled using propensity scores.

‡The results presented in this table differ from those in Figures 1A and 1B because four studies lacking smoking duration data were excluded: CLUE II: Campaign against Cancer and Heart Disease (CLUE II), Japan Public Health Centre-based Prospective Studies I and II (JPHC I and JPHC II) and the NIH-AARP Diet and Health Study.

Supplementary Table S6. Multivariable hazard ratios for bladder cancer risk associated with processed meat and unprocessed red meat consumption among participants with complete data on all smoking variables.

	MEAT INTAKE (grams/day)					
	**<5 (Ref.)**	**5-9.9**	**10-19.9**	**20-39.9**	**≥40**	**Continuous, per 20 g/d increment**
	**n=2,993**	**n=2,054**	**n=2,698**	**n=2,244**	**n=1,094**	**n=11,083**
**Processed meat (23 cohorts)**	**HR (95% CI)**	**HR (95% CI)**	**HR (95% CI)**	**HR (95% CI)**	**HR (95% CI)**	**HR (95% CI)**
Model A + all covariates*	1.00	1.03 (0.97-1.10)	1.07 (1.01-1.13)	1.07 (1.00-1.15)	1.16 (1.06-1.27)	1.05 (1.02-1.07)
Model A + all covariates*+ smoking status	1.00	1.01 (0.95-1.08)	1.04 (0.98-1.10)	1.03 (0.96-1.10)	1.10 (1.01-1.21)	1.03 (1.01-1.05)
Model A + all covariates*+ smoking status + duration	1.00	1.00 (0.95-1.07)	1.03 (0.97-1.09)	1.01 (0.95-1.09)	1.08 (0.98-1.18)	1.03 (1.00-1.05)
Model A + all covariates*+ smoking status + duration + intensity	1.00	1.01 (0.95-1.07)	1.03 (0.97-1.09)	1.01 (0.95-1.09)	1.08 (0.98-1.18)	1.03 (1.00-1.05)
Model A + all covariates*+ smoking status + duration + time since quitting	1.00	1.00 (0.94-1.06)	1.02 (0.96-1.09)	1.01 (0.95-1.09)	1.07 (0.98-1.18)	1.03 (1.01-1.05)
Model A + all covariates*+ smoking status + duration + intensity + time since quitting	1.00	1.00 (0.94-1.06)	1.02 (0.96-1.09)	1.01 (0.95-1.09)	1.07 (0.98-1.18)	1.03 (1.00-1.05)
	**<20 (Ref.)**	**20-39.9**	**40-59.9**	**60-99.9**	**≥100**	**Continuous, per 50 g/day increment**
	**n=2,241**	**n=2,741**	**n=2,344**	**n=2,534**	**n=1,321**	**n=11,181**
**Unprocessed red meat (24 cohorts)**	**HR (95% CI)**	**HR (95% CI)**	**HR (95% CI)**	**HR (95% CI)**	**HR (95% CI)**	**HR (95% CI)**
Model A + all covariates*	1.00	1.04 (0.98-1.10)	1.05 (0.98-1.12)	1.06 (0.99-1.14)	1.09 (0.98-1.21)	1.02 (0.99-1.05)
Model A + all covariates*+ smoking status	1.00	1.03 (0.97-1.09)	1.03 (0.96-1.10)	1.03 (0.96-1.11)	1.05 (0.95-1.16)	1.01 (0.98-1.04)
Model A + all covariates*+ smoking status + duration	1.00	1.01 (0.95-1.08)	1.02 (0.95-1.09)	1.02 (0.94-1.10)	1.03 (0.93-1.14)	1.00 (0.97-1.04)
Model A + all covariates*+ smoking status + duration + intensity	1.00	1.01 (0.95-1.08)	1.02 (0.95-1.09)	1.01 (0.94-1.09)	1.03 (0.93-1.14)	1.00 (0.97-1.04)
Model A + all covariates*+ smoking status + duration + time since quitting	1.00	1.02 (0.96-1.08)	1.02 (0.95-1.09)	1.02 (0.95-1.10)	1.03 (0.93-1.14)	1.00 (0.97-1.04)
Model A + all covariates*+ smoking status + duration + intensity + time since quitting	1.00	1.02 (0.96-1.08)	1.02 (0.95-1.09)	1.02 (0.94-1.10)	1.03 (0.93-1.13)	1.00 (0.97-1.03)

HR, hazard ratio; CI, confidence interval; n, number of bladder cancer cases

*Model A was stratified jointly on baseline age (in years), year of baseline questionnaire completion, and centre (EPIC only). Covariates recorded at baseline were included in multivariable models: race and ethnicity, alcohol drinking, physical activity, processed meat intake or unprocessed red meat intake, total fruit (including fruit juice) intake, total vegetable (including vegetable juice) intake and total energy intake. These variables were entered directly into the multivariable model or, for studies with less than 200 cases, these variables were modelled using propensity scores.

Smoking status categorised as never, former, current; other smoking variables were categorised into quartiles. Data were included only for participants with < 10% missing data on all smoking variables. The analyses included only the following studies: BCDDP, BWHS, CARET, CNBSS, CPS II, CTS, EPIC (female-only), HPFS, IWHS, MCCS, MEC, NHS, NHS II, NLCS, NYSC, PLCO, SCHS, SMC, SMHS, SWHS, WHI and WLHS.

Supplementary Table S7. Multivariable hazard ratios for bladder cancer risk associated with processed meat and unprocessed red meat consumption, restricted to participants in cohorts of teachers and health professionals and those with more than a high school education.

	**Cohorts of teachers and health professionals #**		**Participants with more than a high school education**	
	No. of cases	***HR (95% CI) ****	***No. of cases***	***HR (95% CI) ****
Processed meat				
<5 g/day (Ref.)	692	1.00	2,770	1.00
5-9.9 g/day	444	0.98 (0.84-1.14)	2,160	1.01 (0.94-1.09)
10-19.9 g/day	332	0.99 (0.78-1.24)	2,757	1.02 (0.95-1.10)
20-39.9 g/day	135	0.85 (0.70-1.04)	2,400	1.05 (0.98-1.14)
≥40 g/day	31	1.12 (0.77-1.63)	1,409	1.13 (1.04-1.23)
Per 20 g/day increment	1,624	0.97 (0.88-1.08)	11,496	1.03 (1.00-1.05)
Unprocessed red meat				
<20 g/day (Ref.)	383	1.00	2,764	1.00
20-39.9 g/day	415	0.94 (0.72-1.24)	3,037	1.04 (0.97-1.11)
40-59.9 g/day	195	0.97 (0.79-1.18)	2,146	1.04 (0.98-1.11)
60-99.9 g/day	347	1.08 (0.90-1.31)	2,286	1.07 (1.00-1.15)
≥100 g/day	283	1.07 (0.86-1.33)	1,330	1.08 (0.97-1.22)
Per 50 g/day increment	1,623	1.01 (0.94-1.09)	11,563	1.02 (0.99-1.06)

HR, hazard ratio; CI, confidence interval; N, number of bladder cancer cases

* All models were stratified jointly on baseline age (in years), year of baseline questionnaire completion, and centre (EPIC only) and adjusted for race and ethnicity, smoking status and duration, alcohol consumption, physical activity, analysis for processed meat were additionally adjusted for unprocessed red meat and vice visa, total fruit (including fruit juice) intake, total vegetable (including vegetable juice) intake, and total energy intake; these variables were entered directly into the multivariable model or, for studies with less than 200 cases, modelled using propensity scores.

# Studies were included in this model: the California Teachers Study (CTS), Nurses' Health Study (NHS) and NHS II (all females), and the Health Professionals Follow-up Study (HPFS, all males).
